# Evaluating the sampling effect of propensity score matching for reducing selection bias in medical data

**DOI:** 10.3389/fpubh.2026.1747762

**Published:** 2026-02-10

**Authors:** Minji Roh, Sujin Yum, Gihun Joo, Jae-Won Jang, Hyeonseung Im

**Affiliations:** 1 Interdisciplinary Graduate Program in Medical Bigdata Convergence, Kangwon National University, Chuncheon, Republic of Korea; 2 Kangwon National University, Chuncheon, Republic of Korea; 3 Department of Neurology, Kangwon National University Hospital, Chuncheon, Republic of Korea; 4 Department of Computer Science and Engineering, Kangwon National University, Chuncheon, Republic of Korea

**Keywords:** imbalanced data, machine learning, medical data analysis, propensity score matching, selection bias

## Abstract

**Background:**

In real-world medical data, selection bias can significantly impact the performance of machine learning models, potentially leading to distorted outcomes. However, research aimed at mitigating selection bias remains relatively limited.

**Methods:**

In this study, we evaluate the effectiveness of Propensity Score Matching (PSM) in reducing selection bias and assessing its impact on classification performance in imbalanced medical data. Specifically, we apply PSM alongside five undersampling, three oversampling, and three hybrid sampling techniques to three medical datasets: rapidly progressive dementia prediction (ADNI, *n* = 628, events = 51), hypothyroidism prediction (UCI, *n* = 3,772, events = 3,481), and cardiovascular disease prediction (Kaggle, *n* = 253,680, events = 23,893), each exhibiting varying degrees of demographic selection bias. We train and compare six classification models to assess the impact of each resampling technique on model performance. The magnitude of selection bias is quantified using the standardized mean difference (SMD), while model performance is assessed using the Area Under the Receiver Operating Characteristic Curve (AUROC), the Area Under the Precision-Recall Curve (AUPRC), accuracy, precision, recall, F1-score, specificity, calibration curves, Brier score, and decision curve analysis.

**Results:**

The results indicate that PSM reduces SMD within the dataset, maintains stable classification performance, and enhances the internal validity of the model under conditions of limited or moderate demographic imbalance.

**Conclusion:**

These advantages suggest its potential for improving model reliability and facilitating better generalization to external datasets in real-world medical applications. However, in datasets with extreme selection bias or when overly restrictive matching is applied, PSM can degrade model performance, underscoring the importance of choosing strategies that account for dataset characteristics.

## 1 Introduction

With the rapid advancement of technology and data collection, the utilization of real-world data (RWD) has become increasingly important. Although traditional medical research has favored study designs based on randomized clinical trials (RCTs) due to their high reliability, this approach is costly and limited to specific populations, making it challenging to fully represent the general patient population (1, 2). In contrast, RWD is naturally collected from healthcare institutions or individuals, enabling the inclusion of a broader patient population and offering potential applications across various domains. Consequently, the adoption of RWD in medical research has been steadily increasing (3–7). However, the lack of controlled data collection procedures introduces multiple forms of bias that may compromise the reliability and generalizability of ML-based predictions.

In the literature, the term selection bias has been used in multiple, conceptually distinct contexts. In causal inference, selection bias typically refers to bias arising from non-random treatment assignment, conditioning on post-intervention variables, or missing outcomes, and is discussed in the context of estimating causal effects using observational data (8). In contrast, in machine learning, particularly for healthcare applications, selection bias is commonly used to describe a mismatch between the study population and the target population, often resulting from non-representative sampling, inclusion and exclusion criteria, or data availability constraints (9). These two uses of the term arise from different problem settings and lead to different methodological solutions.

In this study, we focus exclusively on the latter setting: selection bias as a sample representativeness and imbalance problem in supervised ML prediction, rather than as a causal inference problem. Specifically, we consider scenarios in which certain demographic or clinical subpopulations are overrepresented or underrepresented in the available training data. Such imbalances can induce systematic differences in covariate distributions between outcome-defined groups, causing ML models to learn distorted decision boundaries and resulting in degraded performance when deployed in real-world clinical settings (10, 11).

One of the major challenges in machine learning is class imbalance, which has led to the development of various methodologies to address the issue (12, 13). Among the most prominent solutions are data-level and algorithm-level approaches. Data-level methods adjust class ratios by augmenting or removing data through resampling techniques. In contrast, algorithm-level methods include recognition-based learning and cost-sensitive learning approaches. Among these, data resampling techniques are widely utilized in the data preprocessing stage as they help mitigate problems caused by class imbalance, improve model performance, and offer a simple yet intuitive solution. Additionally, they are independent of specific learning algorithms and can be applied flexibly (7, 14). Due to these advantages, resampling techniques remain an active area of research. However, because these methods primarily focus on adjusting sample counts, they may not effectively address underlying distortions in covariate distributions within the dataset.

Propensity Score Matching (PSM) offers an alternative perspective by explicitly targeting covariate balance between groups. Traditionally applied in observational studies to address confounding, PSM has increasingly been adopted in ML contexts as a covariate-aware preprocessing strategy (15, 16). When used for outcome-defined group matching, PSM can be interpreted as a structured resampling approach that prioritizes covariate overlap rather than class prevalence alone. However, it remains unclear under which data conditions and matching ratios PSM can reduce selection bias without compromising predictive discrimination, calibration, or clinical utility in ML-based prediction models.

Therefore, the objective of this study is to investigate the role of PSM as a data-level resampling strategy for mitigating sample selection bias in supervised ML prediction. In particular, we examine how the strength of matching interacts with dataset-specific demographic imbalance to influence downstream model behavior. To achieve this, we quantitatively evaluate the effectiveness of PSM as a data-level resampling technique and examine its impact on data imbalance and classification model performance. This work presents a substantially extended version of our earlier study (17), offering broader experimental evaluation and deeper analytical insights to support medical researchers in assessing the appropriateness of PSM for evaluating prediction models applied to diverse patient populations, particularly under limited demographic selection bias.

## 2 Materials and methods

### 2.1 Datasets

This study investigates the effects of sample selection bias and resampling techniques across heterogeneous real-world data settings. Rather than focusing on disease-specific prediction, three datasets were intentionally selected to represent distinct data sources and selection mechanisms commonly encountered in medical AI research. A summary of each dataset is provided in Table 1 and detailed characteristics are reported in Supplementary material S1.

**Table 1:** Description of datasets.

**No**	**Dataset**	**Prevalence rate**	**Total number**	**Features**	**Demographic variables**	**Healthy *N* (%)**	**Patients *N* (%)**	**Imbalance ratio (IR)**
1	RPD	9–30 (32, 33)	628	10	Age, sex, education	577 (91.88)	51 (8.12)	11.31
2	HypoT	0.3–15 (23, 34)	3,772	3	Age, sex^**^	291 (7.71)	3,481 (92.29)	11.96
3	CVD	4–49 (35–37)	253,680	20	Age^***^, sex^***^, education^***^, income^***^	229,787 (90.58)	23,893 (9.42)	9.62

^**^p < 0.01.

^***^p < 0.001.

The RPD dataset was obtained from the Alzheimer's Disease Neuroimaging Initiative (ADNI) database (adni.loni.usc.edu), a research-oriented cohort launched in 2003 as a public–private partnership led by Michael W. Weiner, MD. ADNI aims to assess the progression of mild cognitive impairment (MCI) and early Alzheimer's disease (AD) using MRI, PET, biomarkers, and clinical evaluations, and has since become a widely used resource in neuroimaging and dementia-related research (18–20). As a registry-based research cohort, ADNI is characterized by voluntary participation and structured follow-up, which may introduce selection mechanisms related to sociodemographic factors and healthcare access. Within this dataset, rapidly progressive dementia (RPD), an acute-onset neurodegenerative condition requiring early intervention (21), was identified using Group-Based Trajectory Analysis based on longitudinal trajectories of Clinical Dementia Rating-Sum of Boxes (CDR-SB) scores. Missing values in the RPD dataset were handled using a K-nearest neighbors (KNN) imputation approach to ensure consistency across resampling experiments.

The HypoT dataset was derived from the Hypothyroidism data within the Thyroid Disease Databases in the UCI Machine Learning Repository (22). This dataset represents routine clinical data, where sample inclusion is primarily driven by healthcare-seeking behavior and diagnostic testing during clinical encounters. Hypothyroidism (HypoT) is a condition characterized by reduced thyroid hormone production, leading to impaired physiological function (23). Owing to the nature of routine care data, this dataset reflects institution and practice-dependent selection patterns. Missing values were addressed using a KNN imputation.

The CVD dataset was obtained from a refined version of the 2015 Behavioral Risk Factor Surveillance System (BRFSS), specifically the Heart Disease Health Indicators dataset available on Kaggle (24). BRFSS is a large-scale, population-based health survey conducted via standardized telephone interviews, and the Kaggle-distributed dataset excludes records with missing values. In this dataset, cardiovascular disease (CVD) status was defined by self-reported diagnoses of coronary heart disease (CHD) or myocardial infarction (MI). Compared to the other datasets, the CVD dataset offers broader population coverage and exhibits relatively weaker selection mechanisms, while still retaining the substantial class imbalance typical of large-scale epidemiological data.

Across all datasets, development and evaluation were conducted using internal resampling procedures to enable fair comparison of resampling strategies under consistent selection mechanisms. None of the datasets was considered fully representative of the general population; rather, each dataset was treated as representative of its respective data-generating process and care context. This design enables systematic evaluation of resampling techniques under varying degrees and forms of sample selection bias.

### 2.2 Data resampling techniques

In this study, five undersampling techniques are employed: Random Under Sampling (RUS), Tomek Links (TL), One-Sided Selection (OSS), Edited Nearest Neighbour (ENN), and Neighbourhood Cleaning Rule (NCR). For oversampling, we utilize Random Over Sampling (ROS), Synthetic Minority Over-sampling Technique (SMOTE), and Adaptive Synthetic Sampling (ADASYN). Additionally, SMOTE-Tomek Links (SMOTE-TL), SMOTE-ENN, and Over-sampling using Propensity Scores (OUPS) (25) are implemented as hybrid sampling techniques.

Undersampling reduces the number of majority class instances to balance the class distribution, thereby lowering computational costs. However, this approach carries the risk of discarding potentially informative samples. In contrast, oversampling increases the number of minority class instances, eliminating the possibility of information loss but potentially introducing artificial instances that may distort model outcomes. Hybrid sampling combines both techniques, enhancing the model's generalizability. However, it also increases the risk of overfitting (26) and is typically applicable only to binary classification tasks.

### 2.3 Propensity score matching

#### 2.3.1 Propensity score modeling and matching procedure

In this study, PSM was employed not as a causal inference framework, but as a covariate balance-oriented resampling strategy to reduce systematic differences in observed covariates between outcome-defined groups before training prediction models. This usage is conceptually closer to case-control matching commonly adopted in medical prediction studies than to treatment-effect estimation.

Let *T* denote a binary group indicator defined by the outcome variable and let *X* denote a vector of observed covariates. The propensity score is defined as the conditional probability of belonging to the outcome-positive group given the covariates,


$$\begin{array}{l} e\left(X\right)=\mathrm{Pr}\left(T=1|X\right), \end{array}$$ (1)

where *T* = 1 indicates individuals with the target condition and *T* = 0 indicates those without the condition. Importantly, in our setting, *T* does not represent an intervention or treatment assignment, and the goal of PSM is not to estimate causal effects but to construct covariate-balanced subsets of cases and controls.

The definition of *T* and the covariates included in the propensity-score model differed across datasets, reflecting data availability and clinical relevance. In the RPD dataset, *T* = 1 indicated individuals with the target outcome, and the propensity score model included age, sex, and education. In the HypoT dataset, *T* = 1 indicated outcome-negative individuals, and age and sex were included as covariates. In the CVD dataset, *T* = 1 indicated individuals with outcome-positive individuals, and the model included age, sex, education, and income.

For all datasets, propensity scores were estimated using logistic regression with main effects only, without interaction terms or non-linear transformations:


$$\text{logit}\left\{e\left(X_{i}\right)\right\}=\beta_{0}+\underset{k}{\sum }\beta_{k}X_{ik}.$$

This specification was chosen for consistency across datasets and to reflect common practice in medical PSM applications.

Matching was conducted using the MatchIt package in R with nearest neighbor matching without replacement. The logit of the propensity score was used as the distance metric (distance = ~logit~). To examine the effect of different matching stringencies, two matching ratios were applied: 1:1 and 1:4. No caliper restriction was imposed, and all other hyperparameters were left at their default values in MatchIt. The same matching procedure and hyperparameter settings were applied consistently across all datasets.

After matching, the resulting subsets were used as inputs for subsequent machine-learning classifiers. Covariate balance before and after matching was evaluated using standardized mean differences (SMDs) for each covariate, as well as summary measures such as the mean and maximum absolute SMD.

#### 2.3.2 Conceptual role of PSM as a resampling strategy

Conceptually, PSM modifies the training data in a fundamentally different manner from standard class-imbalance techniques. While under-sampling and over-sampling methods primarily manipulate class prevalence to address imbalance, they typically operate without explicitly considering the joint distribution of observed covariates. In contrast, PSM directly targets covariate balance by restricting the analysis to regions of sufficient overlap in the propensity score space between outcome-defined groups.

As a consequence, PSM may alter both covariate distributions and class prevalence indirectly, with the final sample composition determined by the availability of suitable matches rather than by a predefined class ratio. In this study, we therefore interpret PSM not as an outcome-driven balancing technique, but as a structured, covariate-aware resampling strategy that complements conventional resampling methods commonly used in supervised classification.

### 2.4 Classification models

To compare classification performance, we employ common machine learning classification models, including Logistic Regression, Random Forest (RF), eXtreme Gradient Boosting (XGBoost), Light Gradient Boosting Model (LGBM), Naïve Bayes (NB) classifier, and the Soft Voting ensemble model. Detailed hyperparameter configurations for each classification model are provided in Supplementary material S2.

- Linear model: Logistic Regression
- Tree-based models: RF, XGBoost, LGBM
- Probability-based model: Naïve Bayes
- Voting-based model: Soft Voting

### 2.5 Evaluation metrics

Model performance was assessed through distributional balance measures and predictive metrics at both fixed and varying decision thresholds.

To assess distributional balance between groups, we calculated the Standardized Mean Difference (SMD) and the Imbalance Ratio (IR). The SMD is calculated as the difference in covariate-specific means between two groups, divided by the pooled standard deviation of the corresponding covariate (27). Since it is a standardized measure of mean differences for each covariate, it can be used to assess covariate imbalance between groups, which is commonly reported in studies addressing selection bias (28). A value below 0.2 is considered a small effect size, whereas a value of 0.8 or higher is regarded as a large effect size (29). The IR was computed as the ratio of the resampled size of the original majority class to that of the original minority class. Thus, IR = 1 indicates perfect balance; IR >1 indicates the baseline-majority class remains larger; and IR < 1 indicates a class-size reversal where the baseline-minority class becomes larger.

Predictive performance was evaluated using both threshold-dependent and threshold-independent metrics. Threshold-dependent evaluation was conducted using accuracy, precision, recall, F1-score, and specificity, which quantify classification performance at a standard probability threshold of 0.5. These metrics provide complementary perspectives on model behavior, particularly in imbalanced classification settings.

Discrimination and calibration were assessed using threshold-independent metrics and visual tools. AUROC summarizes sensitivity-specificity tradeoffs across all thresholds through ROC curves. AUPRC, visualized through precision-recall curves, is particularly informative for imbalanced datasets. The Brier score quantifies probabilistic accuracy by measuring mean squared error between predicted probabilities and observed outcomes. Calibration plots were used to assess the agreement between predicted probabilities and observed event rates. Decision curve analysis (DCA) evaluated clinical utility by quantifying net benefit across varying risk thresholds. Detailed descriptions of all evaluation metrics are provided in Supplementary material S3.

The experiments were conducted using Python 3.10.12, including numpy 1.25.2, pandas 2.0.3, scikit-learn 1.2.2, imbalanced-learn 0.10.1, xgboost 2.0.3, lightgbm 4.1.0, and smote-variants 0.7.3, as well as R 4.3.3 with MatchIt 4.5.5.

This study has ethical approval, consent to participate, and consent for publication, and was approved by the Institutional Review Board of Kangwon National University (IRB No. KWNUIRB-2023-10-001).

## 3 Results

In this study, we applied various resampling techniques and PSM (1:4 and 1:1) to the RPD, HypoT, and CVD datasets and compared the SMD of the datasets as well as the classification performance of machine learning models (LR, RF, LGBM, XGBoost, NB, and the Soft Voting ensemble model). The datasets were min-max normalized, and five-fold cross-validation was employed.

To account for the stochasticity introduced by resampling procedures and cross-validation, all experiments were repeated using 10 different random seeds. Preliminary analyses indicated that model performance metrics stabilized within this range, with only marginal variance reduction observed beyond 10 repetitions. Given the modest performance differences among methods and the computational cost associated with additional runs, 10 repetitions were considered sufficient to provide robust and comparable estimates. All resampling techniques were applied without hyperparameter tuning. For PSM, in addition to 1:1 matching, we also employed 1:4 matching to prevent excessive information loss and mitigate the effects of incomplete matching.

For each dataset, a representative model was selected based on the mean AUPRC aggregated across all resampling strategies using out-of-fold predictions from 10 repetitions of five-fold cross-validation. This selection criterion was pre-specified to reflect the primary discrimination objective under class imbalance and to identify a stable reference model for subsequent analyses. Using the selected model, we further examined the effects of different resampling and PSM strategies on model behavior, rather than to claim optimal performance. The specific models selected according to this criterion are summarized in Supplementary material S4.

When evaluating classification performance, additional experiments were conducted under the same conditions, excluding demographic variables, to assess the degree of information loss introduced by PSM.

### 3.1 Effect of resampling techniques on selection bias reduction

To compare the impact of different resampling techniques on selection bias in demographic variables, we compute the SMD and summarize the results in Table 2.

**Table 2:** Comparison of the standardized mean difference (SMD) by datasets, variables, and resampling techniques.

**Category**	**Technique**	**RPD**			**HypoT**		**CVD**			
		**Age**	**Sex**	**Education**	**Age**	**Sex**	**Age**	**Sex**	**Education**	**Income**
Baseline		0.10 ± 0.07	0.08 ± 0.05	0.14 ± 0.09	0.03 ± 0.02	0.20 ± 0.03	0.87 ± 0.00	0.30 ± 0.00	0.33 ± 0.00	0.47 ± 0.00
Undersampling	RUS	0.20 ± 0.14	0.14 ± 0.11	0.17 ± 0.12	0.05 ± 0.03	0.19 ± 0.07	0.87 ± 0.01	0.30 ± 0.01	0.33 ± 0.01	0.47 ± 0.01
	TL	0.12 ± 0.07	0.09 ± 0.06	0.13 ± 0.09	0.03 ± 0.02	0.20 ± 0.03	0.89 ± 0.00	0.30 ± 0.00	0.34 ± 0.00	0.49 ± 0.00
	OSS	0.11 ± 0.07	0.08 ± 0.06	0.13 ± 0.09	0.10 ± 0.06	0.20 ± 0.03	0.89 ± 0.00	0.30 ± 0.00	0.34 ± 0.00	0.49 ± 0.00
	ENN	0.15 ± 0.07	0.12 ± 0.07	0.16 ± 0.09	0.03 ± 0.02	0.20 ± 0.03	1.05 ± 0.00	0.33 ± 0.00	0.40 ± 0.00	0.57 ± 0.00
	NCR	0.15 ± 0.07	0.12 ± 0.07	0.16 ± 0.09	0.03 ± 0.02	0.20 ± 0.03	1.01 ± 0.00	0.31 ± 0.00	0.38 ± 0.00	0.55 ± 0.00
Oversampling	ROS	0.10 ± 0.07	0.08 ± 0.05	0.12 ± 0.09	0.03 ± 0.02	0.19 ± 0.04	0.87 ± 0.00	0.30 ± 0.00	0.33 ± 0.00	0.47 ± 0.00
	SMOTE	0.11 ± 0.08	0.13 ± 0.08	0.19 ± 0.11	0.03 ± 0.02	0.20 ± 0.04	0.89 ± 0.00	0.32 ± 0.00	0.32 ± 0.00	0.47 ± 0.01
	ADASYN	0.09 ± 0.06	0.13 ± 0.07	0.19 ± 0.12	0.09 ± 0.04	0.23 ± 0.04	0.84 ± 0.00	0.30 ± 0.00	0.30 ± 0.00	0.44 ± 0.00
Hybrid sampling	SMOTE-TL	0.12 ± 0.08	0.14 ± 0.08	0.19 ± 0.11	0.03 ± 0.02	0.21 ± 0.04	0.89 ± 0.00	0.32 ± 0.00	0.32 ± 0.00	0.47 ± 0.00
	SMOTE-ENN	0.21 ± 0.10	0.24 ± 0.11	0.23 ± 0.12	0.03 ± 0.02	0.21 ± 0.04	1.22 ± 0.01	0.35 ± 0.00	0.48 ± 0.00	0.68 ± 0.01
	OUPS	0.18 ± 0.09	0.06 ± 0.05	0.15 ± 0.09	0.06 ± 0.04	0.26 ± 0.04	0.89 ± 0.01	0.28 ± 0.01	0.39 ± 0.01	0.53 ± 0.01
PSM	PSM1:4	0.04 ± 0.03	0.05 ± 0.04	0.03 ± 0.03	0.01 ± 0.00	0.00 ± 0.00	0.07 ± 0.00	0.10 ± 0.00	0.06 ± 0.00	0.05 ± 0.00
	PSM1:1	0.08 ± 0.06	0.11 ± 0.10	0.06 ± 0.06	0.00 ± 0.00	0.00 ± 0.00	0.00 ± 0.00	0.00 ± 0.00	0.00 ± 0.00	0.00 ± 0.00

Among the undersampling techniques, TL and OSS produce results similar to the baseline. However, RUS, ENN, and NCR generally tend to increase selection bias. Notably, for the Age variable in the CVD dataset, the SMD increases significantly to 0.18 when ENN is applied.

Among the oversampling techniques, ROS has minimal impact on selection bias, whereas SMOTE-based techniques (SMOTE, ADASYN, SMOTE-TL, and SMOTE-ENN) exhibit a general trend of increasing the SMD.

In contrast, PSM (1:1 and 1:4) successfully reduces the SMD to below 0.01 across all variables and demonstrates a stable bias correction effect, as confirmed by standard deviation comparisons.

### 3.2 Results of resampling for class balancing

**Figure 1:**
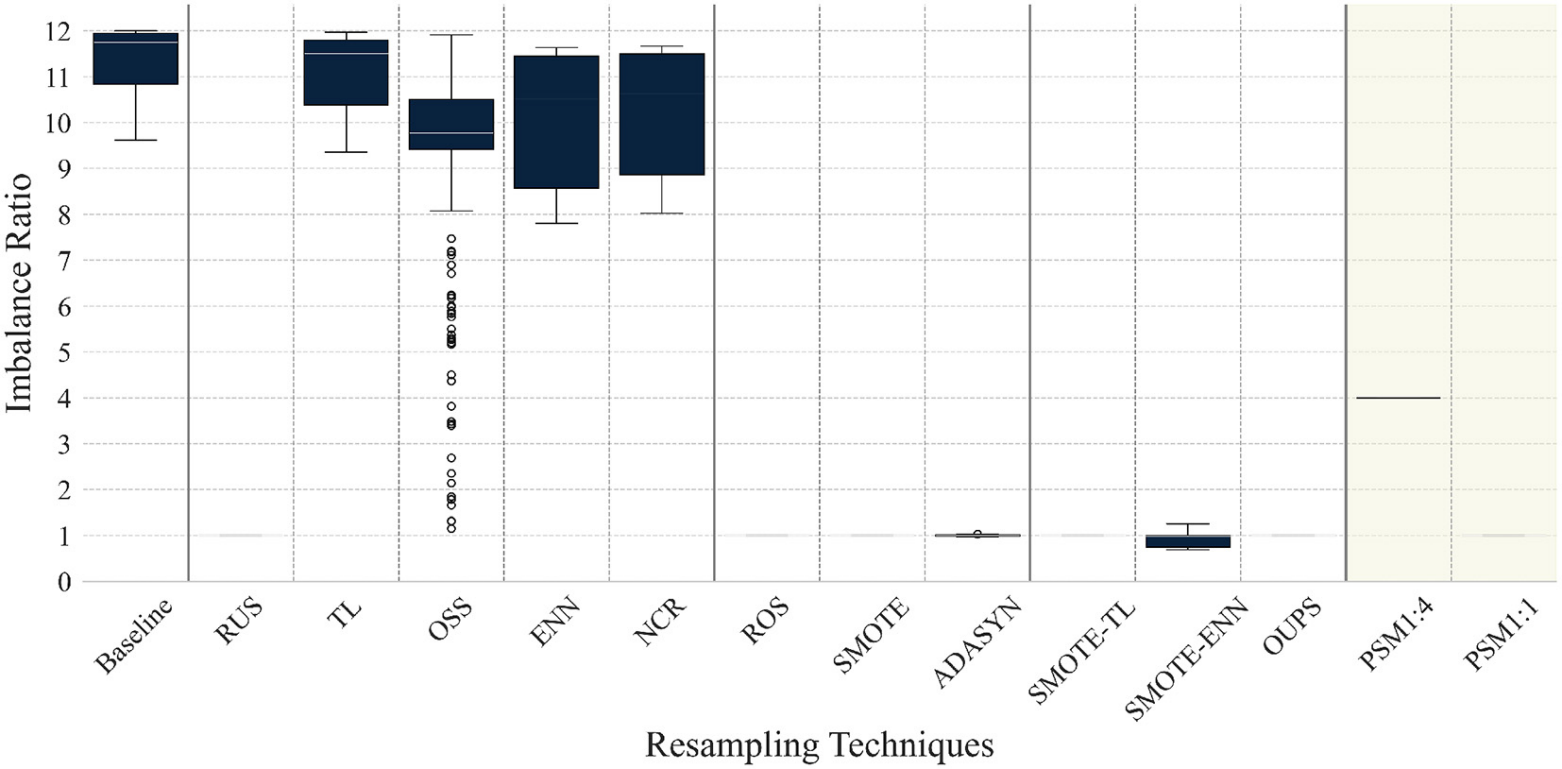
Imbalance ratio (IR) distribution by resampling techniques. IR is defined as the ratio of the larger to the smaller class size, with IR = 1 indicating a balanced class distribution.

**Figure 2:**
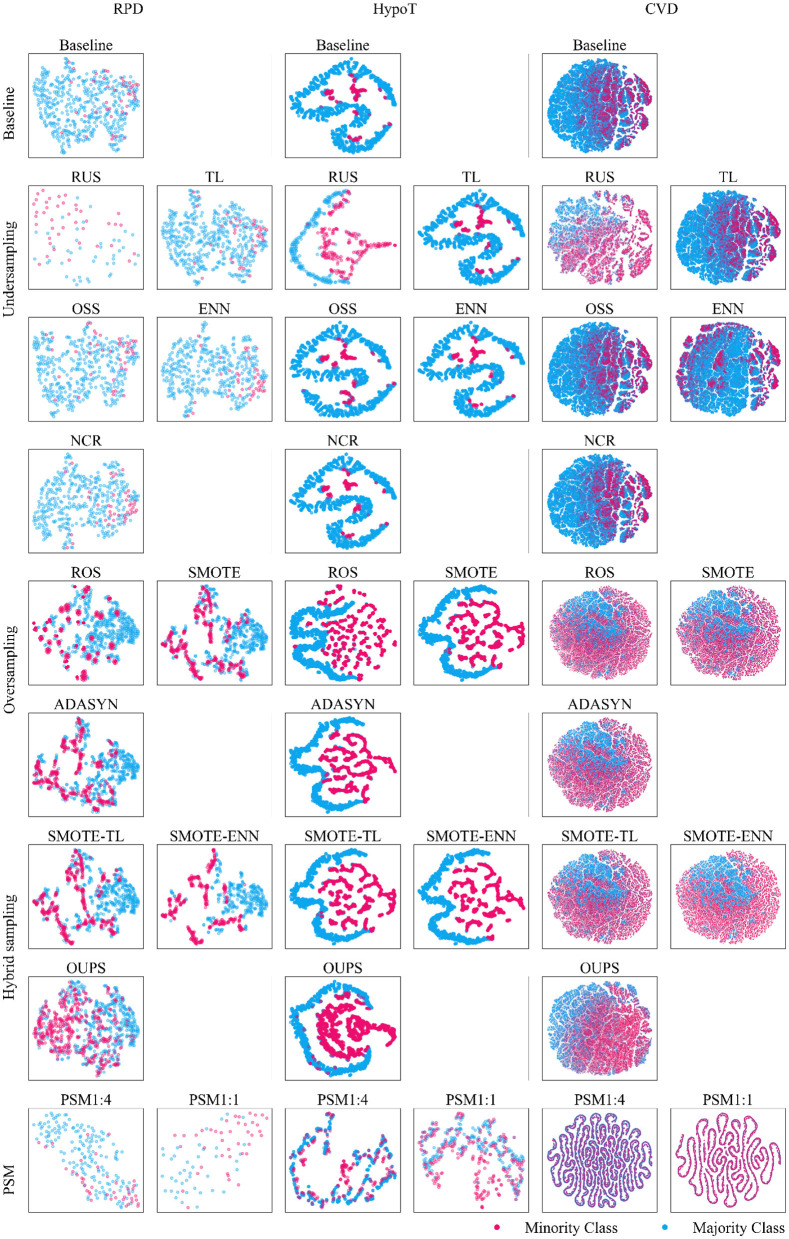
t-SNE visualization of different resampling techniques. Due to differences in sample sizes, RPD and HypoT were plotted using an alpha of 0.4 and a size of 20. For CVD, an alpha of 0.1 and a size of 1 were generally used, but for oversampling and hybrid sampling within CVD, which involved larger sample sizes, a smaller alpha of 0.05 and a size of 0.1 were used to avoid overplotting.

Figure 1 presents the IR observed after applying each resampling technique and PSM, where the results are shown for all datasets. Figure 2 provides the t-SNE visualization of the datasets after resampling and PSM. Detailed numerical results are provided in Supplementary material S5.

Randomized resampling techniques (RUS and ROS) and SMOTE-based techniques (SMOTE, SMOTE-TL, and OUPS) achieve an IR of 1, ensuring an equal distribution of majority and minority class instances without requiring hyperparameter tuning. In contrast, certain undersampling techniques, such as TL, ENN, and NCR are less effective in mitigating class imbalance. OSS exhibits high variability, with IR values ranging from a minimum of 1.15 to a maximum of 11.91. We note that undersampling techniques can significantly reduce the dataset size when applied to highly imbalanced data. For example, for RUS, the majority class is reduced to match the minority class at a 1:1 ratio, reducing the final sample size to 82 (RPD), 466 (HypoT), and 38,230 (CVD), compared to 502, 3,017, and 202,944 in the baseline, respectively. Among the hybrid sampling techniques, SMOTE-ENN (IR 0.91 ± 0.15) is generally effective in reducing class imbalance. However, it is the only method where the majority class becomes smaller than the minority class in certain cases. This behavior can be attributed to the hybrid nature of SMOTE-ENN, which simultaneously applies minority oversampling and majority undersampling, occasionally leading to an overcorrection of class imbalance relative to the baseline distribution.

### 3.3 Classification performance by datasets and resampling techniques

Figure 4 illustrates the classification performance across datasets and resampling techniques, comparing models trained with and without demographic variables. Figures 4–6 further present these results separately for each dataset.

**Figure 3:**
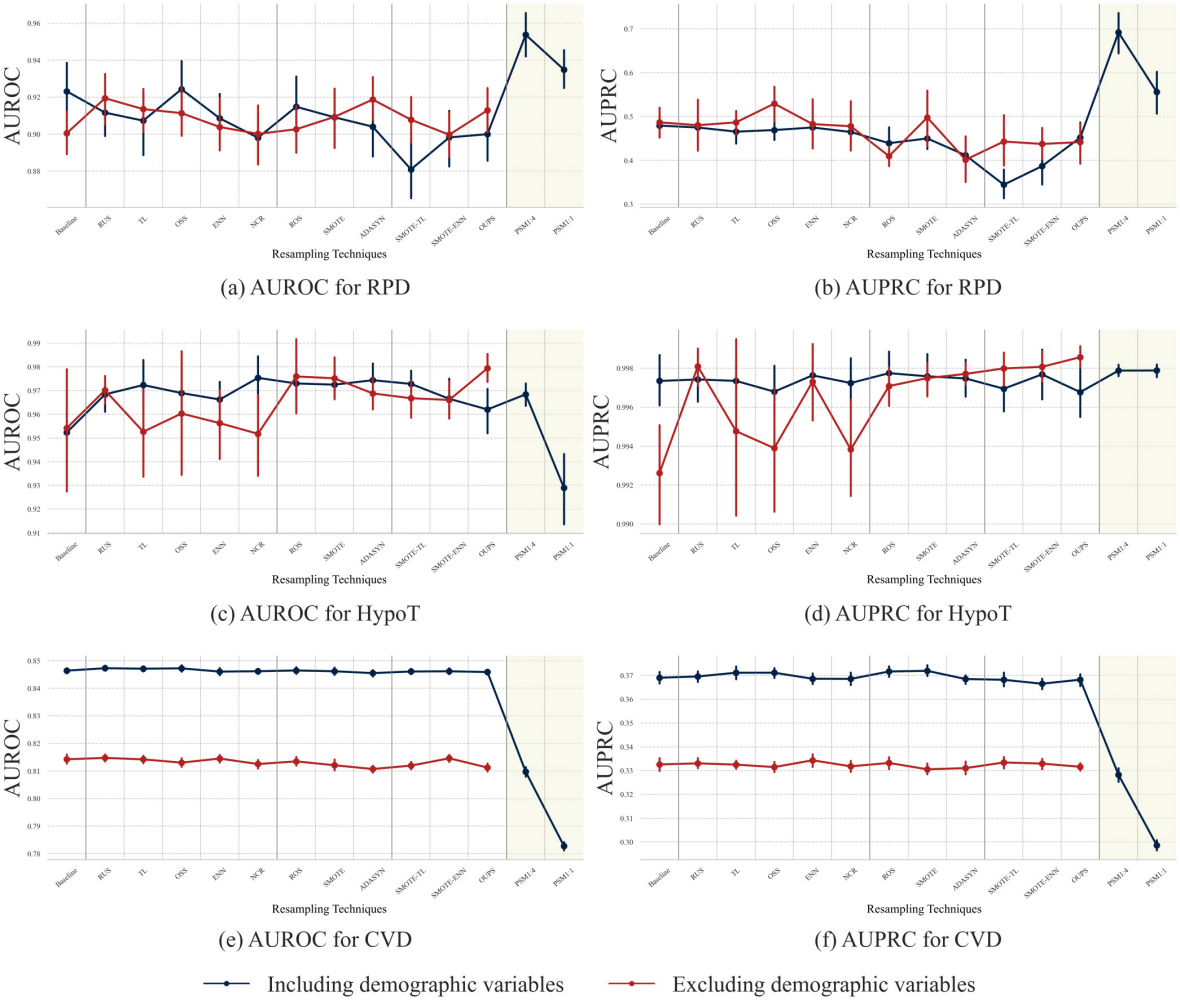
Classification performance by resampling techniques with and without demographic variables for each dataset. As PSM inherently incorporates demographic variables into its matching process, results for the “excluding demographics" setting are not reported for PSM.

In the RPD dataset, the difference in AUROC when demographic variables are included versus excluded remains relatively small, with a maximum deviation of 0.0126 in the mean AUROC and 0.0284 in the mean AUPRC. Additionally, the trend in performance variation due to resampling remains consistent regardless of the presence of demographic variables (Figure 3).

**Figure 4:**
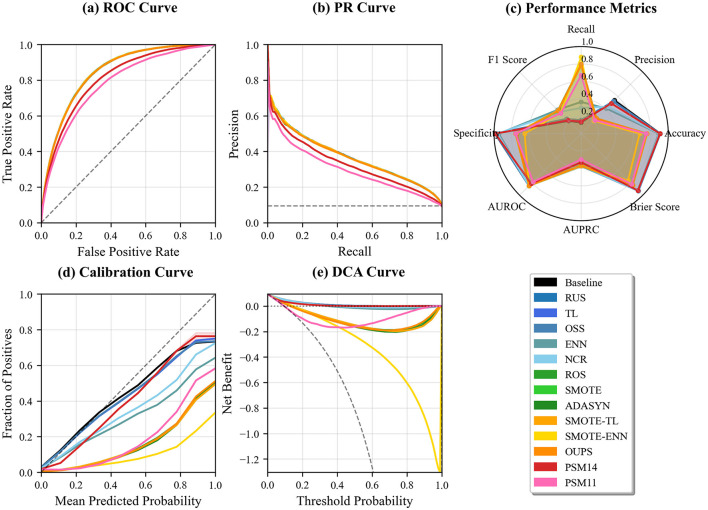
Comprehensive performance comparison of resampling techniques on the RPD dataset, evaluated in terms of discrimination, calibration, and clinical utility. Panels show **(a)** ROC curves, **(b)** PR curves, **(c)** aggregated classification metrics, **(d)** calibration curves, and **(e)** decision curve analysis (DCA).

Figure 4 indicates that, under models including demographic variables, the relative performance ordering across resampling techniques is largely preserved. PSM1:4 achieves the highest discriminative performance in both ROC and PR curve analyses, followed by PSM1:1. Furthermore, PSM1:4 demonstrates consistently strong performance across the ROC curve (Figure 4a), PR curve (Figure 4b), and aggregated performance metrics (Figure 4c). PSM1:1 shows the second-best discriminative performance in terms of ROC Curve and PR Curve (Figures 4a, b), but exhibits relatively lower values in several aggregated performance metrics compared with several non-PSM resampling strategies (Figure 4c). However, calibration and decision curve analyses reveal pronounced deviations and unstable net benefit for PSM1:1, whereas PSM1:4 remains well calibrated (Figure 4d) and provides stable clinical net benefit across a wide range of threshold probabilities (Figure 4e).

**Figure 5:**
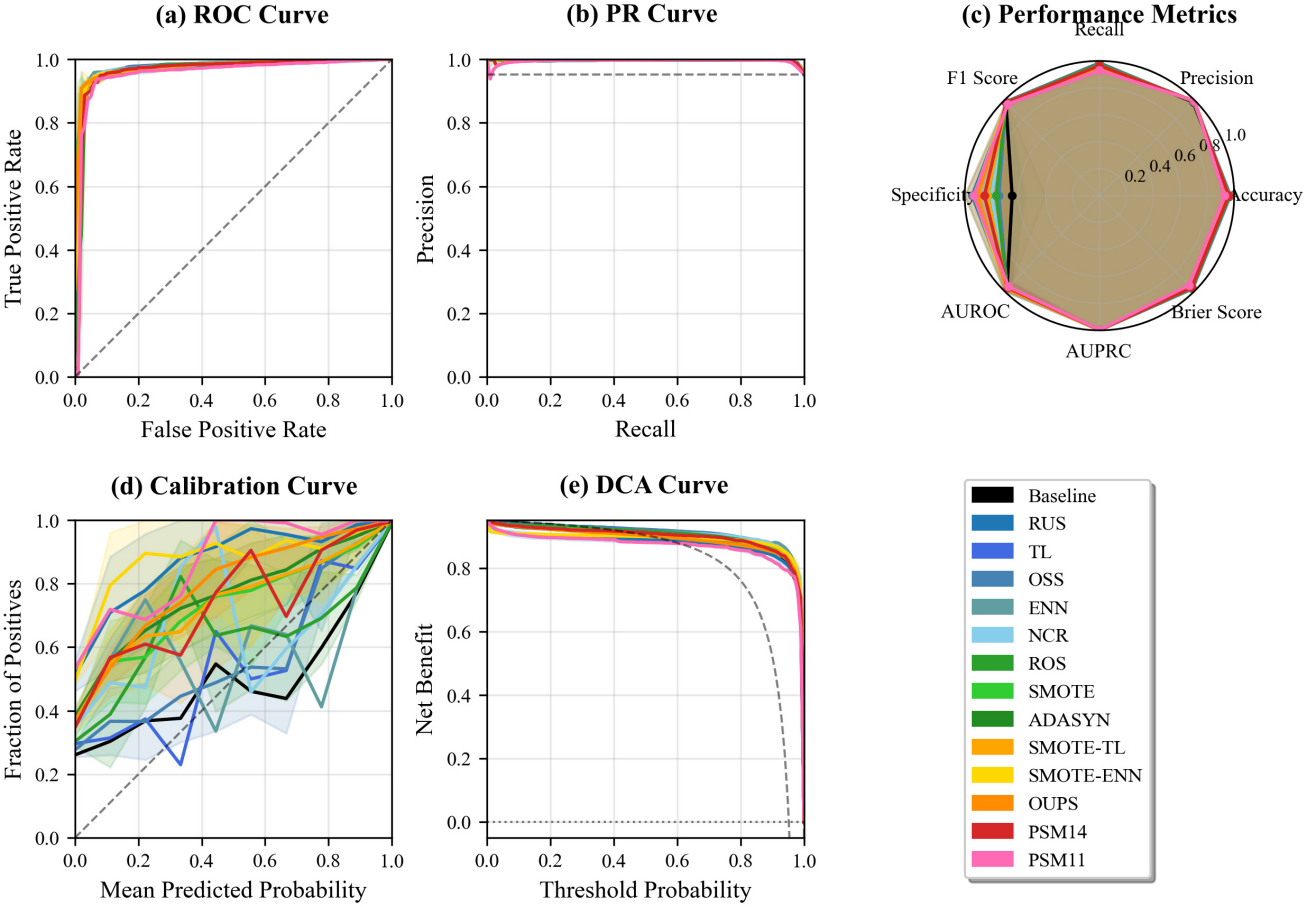
Comprehensive performance comparison of resampling techniques on the HypoT dataset, evaluated in terms of discrimination, calibration, and clinical utility. Panels show **(a)** ROC curves, **(b)** PR curves, **(c)** aggregated classification metrics, **(d)** calibration curves, and **(e)** decision curve analysis (DCA).

In the HypoT dataset, classification performance improves across all techniques when demographic variables are removed. However, when demographic variables are included, AUROC and AUPRC already exhibit high baseline values, limiting the potential for further gains from resampling. The largest improvements are observed for PSM1:4 and PSM1:1, though these remain modest (Figure 3). Figure 5 further illustrates this ceiling effect. Both ROC and PR curves (Figures 5a, b) show that all resampling techniques cluster tightly near the upper-left and upper-right regions, respectively, indicating near-optimal discrimination performance. Differences among methods are minimal, and the baseline model also maintains strong performance. In terms of aggregated performance metrics, PSM-based approaches achieve performance levels comparable to other resampling techniques. Notably, specificity under PSM is lower than that of some undersampling methods but remains higher than that achieved by oversampling approaches (Figure 5c). Calibration curves (Figure 5d) demonstrate that most techniques closely follow the ideal diagonal. Decision curve analysis (Figure 5e) shows uniformly high net benefit across a wide range of thresholds, suggesting that resampling provides limited additional clinical utility in this high-performing scenario.

**Figure 6:**
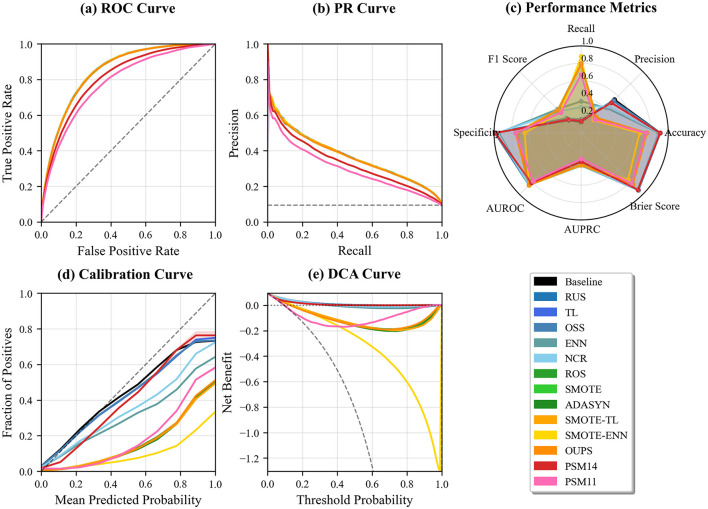
Comprehensive performance comparison of resampling techniques on the CVD dataset, evaluated in terms of discrimination, calibration, and clinical utility. Panels show **(a)** ROC curves, **(b)** PR curves, **(c)** aggregated classification metrics, **(d)** calibration curves, and **(e)** decision curve analysis (DCA).

For the CVD dataset, classification performance is generally higher when demographic variables are included. Under this setting, most non-PSM resampling techniques achieve comparable or improved performance, whereas PSM1:4 and PSM1:1 exhibit a notable decline and remain inferior to other approaches even when demographic variables are excluded (Figure 3). Figure 6 further illustrates these differences across resampling techniques, including demographic variables. The ROC curves (Figure 6a) indicate that all resampling techniques outperform the random baseline, with non-PSM resampling techniques achieving higher true positive rates across a wide range of false positive rates. In contrast, PSM-based approaches show heterogeneous behavior, with PSM1:1 consistently underperforming and PSM1:4 showing relatively stable discrimination only in intermediate false positive rate regions. These patterns are more pronounced in the PR curves (Figure 6b), where non-PSM resampling techniques generally maintain higher precision across recall levels. The aggregated performance metrics indicate substantial trade-offs across resampling strategies (Figure 6c). PSM1:4 exhibits a pattern similar to undersampling techniques, whereas PSM1:1 shows performance trends more closely aligned with hybrid sampling methods, reflecting differing balances among discrimination, calibration, and clinical utility. Calibration and decision curve analyses (Figures 6d, e) further highlight substantial differences in probability estimation and clinical utility, with PSM1:4 remaining relatively well calibrated and clinically stable, whereas PSM1:1 provides limited net benefit.

Detailed numerical results for all experimental settings, as well as comprehensive performance comparisons excluding demographic variables for each dataset, are provided in Supplementary material S6.

### 3.4 Comparison of classification performance based on variable distribution

**Figure 7:**
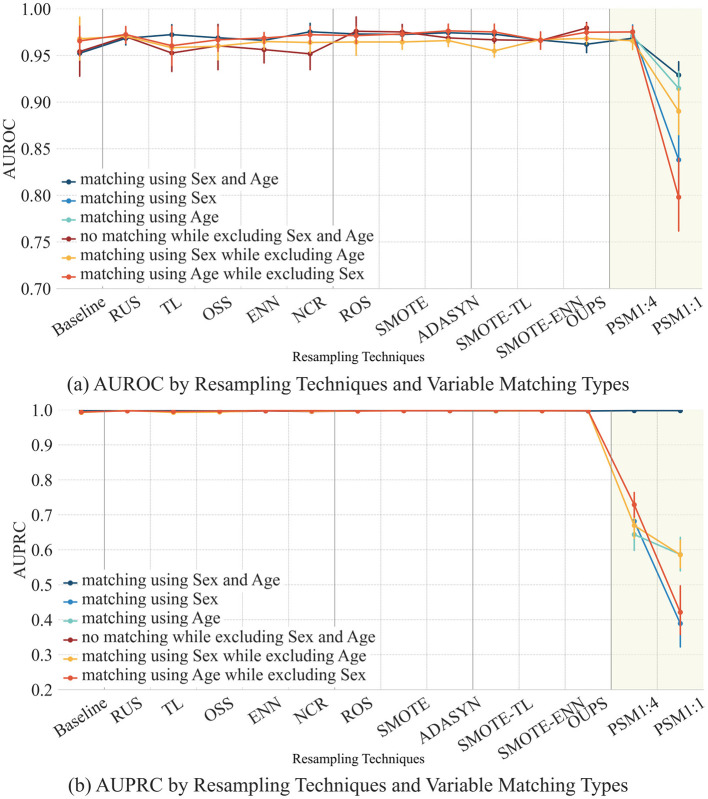
Classification performance by resampling techniques based on variable distribution. As propensity score matching (PSM) directly utilizes demographic variables during matching, results for the “no matching while excluding Sex and Age” setting are not reported, unlike the other resampling techniques. Instead, PSM enables variable-specific matching, and results for matching by Sex and Age are presented separately. Panels show **(a)** AUROC and **(b)** AUPRC across resampling methods when matching is performed using sex, age, or both variables. Because propensity score matching (PSM) explicitly relies on the selected covariates for matching, a “no matching” condition does not apply to PSM and is therefore not reported. Instead, PSM results are presented separately according to the variables included in the matching process, enabling direct comparison of variable-specific matching effects.

In the HypoT dataset, among the two demographic variables, Age does not exhibit statistically significant differences between classes, whereas Sex does. To examine the effect of each variable, PSM is performed under four conditions: (1) matching using Age only; (2) matching using Sex only; (3) matching using Age while excluding Sex; and (4) matching using Sex while excluding Age. The distributions of these matched variables are presented in Table 3, and the classification performance under each condition is shown in Figure 7.

**Table 3:** Comparison of 4:1 matched patients and controls based on matching variables in the HypoT dataset.

**Matching variables**	**Post-matching variables**	**Patients *N* (%)**	**Controls *N* (%)**	***p*-value**	**SMD**
Baseline	*N*	3,481	291		
	Age	51.76 (20.17)	51.52 (19.11)	0.842	0.012
	Sex				
	Male	1077 (30.9)	65 (22.3)	0.003^**^	0.196
	Female	2404 (69.1)	226 (77.7)		
	TSH	2.17 (10.21)	39.23 (74.38)	< 0.001^***^	0.698
Sex, age	*N*	932	233		
	Age	51.65 (18.55)	51.59 (18.77)	0.967	0.003
	Sex				
	Male	220 (23.6)	55 (23.6)	1.000	< 0.001
	Female	712 (76.4)	178 (76.4)		
	TSH	2.09 (6.97)	37.62 (68.69)	< 0.001^***^	0.728
Sex	*N*	932	233		
	Age	51.94 (18.83)	52.29 (18.65)	0.799	0.019
	Sex				
	Male	224 (24.0)	56 (24.0)	1.000	< 0.001
	Female	708 (76.0)	177 (76.0)		
	TSH	2.30 (13.69)	38.63 (75.33)	< 0.001^***^	0.671
Age	*N*	932	233		
	Age	52.35 (18.46)	52.29 (18.65)	0.967	0.003
	Sex				
	Male	289 (31.0)	56 (24.0)	0.045^*^	0.157
	Female	643 (69.0)	177 (76.0)		
	TSH	2.02 (4.92)	38.63 (75.33)	< 0.001	0.686

TSH, thyroid-stimulating hormone.

^*^p < 0.05.

^**^p < 0.01.

^***^p < 0.001.

The results in Table 3 indicate that selection bias is effectively mitigated when both variables are used for matching, as well as when Sex alone is used. However, when matching is performed using Age alone, Sex-based selection bias is not fully corrected.

As shown in Figure 7, AUROC and AUPRC remain largely stable across most resampling techniques regardless of the matching strategy, except for PSM-based approaches. When all demographic variables are excluded, classification performance is generally highest, whereas both PSM1:4 and PSM1:1 exhibit a noticeable decline. Moreover, removing Sex while performing matching using Age consistently results in lower performance than removing Age and matching using Sex, highlighting the greater predictive contribution of Sex.

Within the PSM-based methods, PSM1:4 demonstrates relatively stable performance across matching conditions, while PSM1:1 shows more pronounced degradation. Supplementary analyses of calibration curves and decision curve analysis (Supplementary material S6; Supplementary Figures S4–S7) further support these findings, showing that PSM1:4 maintains more stable calibration and achieves higher net benefit, whereas PSM1:1 exhibits poorer calibration and substantially reduced net benefit. Overall, these results indicate that PSM1:4 provides a more consistent balance between discrimination, calibration, and clinical utility in the HypoT dataset.

## 4 Discussion

In this study, PSM is applied as a data-level resampling technique to correct selection bias and evaluate its impact on the performance of machine learning classification models.

### 4.1 Advantages of propensity score matching in mitigating selection bias

The experimental results indicate that PSM reduces selection bias more effectively than other resampling techniques. Across all datasets, PSM lowers the SMD of demographic variables to values close to zero and yields the smallest standard deviation.

Traditional resampling techniques primarily focus on class balancing. They adjust the overall data distribution but do not consider the distribution of other variables. In contrast, PSM matches samples while considering both specific variables and class labels, making it a more effective method for mitigating selection bias.

A statistical analysis using chi-square tests and mean comparisons shows that variables with statistically significant differences in the original dataset exhibit higher *p*-values after PSM. This suggests that PSM effectively reduces selection bias.

PSM allows researchers to explicitly specify variables for matching, leading to a more stable reduction of bias in targeted variables. Consequently, PSM enhances the internal validity of the dataset, making it a valuable tool for bias correction.

### 4.2 Classification performance of PSM

Our results demonstrate that the effectiveness of PSM is highly dependent on the underlying distributional characteristics of the dataset. In the RPD dataset, where none of the demographic variables exhibit statistically significant differences between classes, PSM consistently achieves competitive or superior classification performance compared with other resampling techniques. Across discrimination, calibration, and decision curve analyses (Figure 4), PSM-based approaches, particularly PSM1:4, show stable performance, indicating that matching can mitigate residual selection bias without substantially distorting informative feature distributions. The superior performance of PSM1:4 relative to PSM1:1 suggests that a higher matching ratio provides additional training samples while preserving sufficient covariate balance.

In the HypoT dataset, where only a subset of demographic variables exhibits moderate imbalance, classification performance remains largely comparable, with only a slight decline observed. Although PSM1:4 and PSM1:1 achieve classification performance comparable to that obtained after removing demographic variables, Figure 5 reveals that overall discrimination is near saturation. ROC and PR curves cluster tightly across all resampling strategies, and both calibration and decision curve analyses indicate uniformly high probability reliability and clinical net benefit. These findings suggest that, in datasets with intrinsically high separability, resampling, including PSM, provides limited additional benefit beyond the baseline model.

Overall, the impact of PSM varies by dataset and depends on both the degree of demographic imbalance and the matching ratio. In datasets with weak or moderate demographic–outcome associations, such as RPD and HypoT, differences between matching ratios are more evident in calibration and clinical utility than in discrimination. In these settings, PSM1:4 maintains stable discrimination, calibration, and net benefit, whereas PSM1:1 shows consistent degradation across evaluation metrics, indicating that overly restrictive matching can compromise model reliability even when discrimination is near saturation.

In contrast, in the CVD dataset, where all variables—including demographic ones—exhibit statistically significant distributional differences between classes (*p*-value < 0.001) with large effect sizes, applying PSM leads to a consistent decline in classification performance. As illustrated in Figure 6, this degradation is evident not only in AUROC and AUPRC but also in calibration behavior and clinical net benefit. In particular, PSM1:1 shows inferior performance across multiple evaluation dimensions, while PSM1:4 exhibits only partial stability. These results indicate that, when predictors themselves encode strong disease-related signals, enforcing covariate balance through matching can attenuate informative heterogeneity and reduce model discriminability.

These findings highlight the importance of conducting preliminary data analysis and strategically selecting preprocessing techniques when developing machine learning models using imbalanced medical datasets collected outside of RCTs. Notably, PSM demonstrates the ability to address both selection bias and class imbalance depending on the distributional characteristics of the dataset. As such, PSM presents a practical and lightweight preprocessing strategy for AI-driven clinical decision-making, offering improvements in data consistency and model performance without requiring major modifications to model architectures.

It has been previously argued that sampling-based imbalance correction techniques may adversely affect probability calibration by altering the underlying data distribution. Such concerns emphasize that improvements in discrimination metrics do not necessarily translate into reliable probabilistic predictions. In this study, we explicitly address this issue by evaluating calibration curves, Brier scores and decision curve analysis across all resampling strategies. Our results demonstrate that the impact of resampling on calibration is highly dataset dependent. While certain oversampling methods exhibit noticeable calibration distortion, particularly in the CVD dataset, PSM-based approaches, especially PSM1:4, maintain relatively stable calibration in datasets with limited or moderate covariate imbalance. These findings suggest that the calibration-related risks associated with sampling techniques are not uniform and should be assessed empirically rather than assumed *a priori*.

### 4.3 Impact of selection bias on classification performance

Our findings indicate that reductions in selection bias do not uniformly translate into improved classification performance. Although bias mitigation is essential for internal validity, aggressive matching may attenuate informative heterogeneity, particularly in datasets with minimal baseline demographic imbalance. This trade-off is evident in the contrasting behavior of PSM1:4 and PSM1:1, with the latter showing greater degradation in discrimination, calibration, and clinical net benefit despite stronger covariate balance.

To quantitatively assess the impact of selection bias on the classification performance of machine learning models, we analyze the correlation between the degree of selection bias after applying each resampling technique and the corresponding classification performance. The results indicate a negative correlation between the mean SMD and classification performance in the RPD and HypoT datasets, whereas a positive correlation is observed in the CVD dataset (Table 4). The absolute values of all correlation coefficients exceed 0.650, indicating a strong correlation. In particular, the CVD dataset exhibits very high correlations of 0.902 and 0.897, suggesting a significant relationship between selection bias and classification performance. These findings confirm that selection bias within a dataset influences the learning process of classification models.

**Table 4:** Correlation between selection bias and classification performance.

**Dataset**	**SMD-AUROC**	**SMD-AUPRC**
RPD	–0.694	–0.815
HypoT	–0.846	–0.650
CVD	0.902	0.897

This study also reconfirms the issue reported in previous research, where selection bias leads to the underestimation of certain group characteristics (30). In the HypoT dataset, the only remaining variable after excluding demographic variables is TSH, which is a direct biomarker of hypothyroidism. As a result, the classification performance remains high using this variable alone. The *p*-value of TSH is below 0.001, indicating a statistically significant difference in distribution between classes.

However, in this experiment, AUROC and AUPRC increase across all resampling techniques when demographic variables are removed. According to previous studies, hypothyroidism is also known to be influenced by age and sex. Thus, the observed decline in performance when sex and age are included suggests that the model fails to accurately capture the patterns in which these demographic factors influence class differentiation. This trend is further illustrated in Figure 7, where an increase in the availability of sex and age information results in a decline in classification performance.

Such a phenomenon deteriorates the internal validity of the model and increases the risk of underdiagnosis or overdiagnosis for certain groups. In contrast, PSM demonstrates classification performance comparable to that of resampling techniques where demographic variables are removed, despite incorporating these variables.

These findings indicate that PSM mitigates the impact of dataset bias, enhances classification performance, and strengthens internal validity, thereby improving the reliability of results and facilitating better generalization to external datasets. Although the effect may vary depending on the characteristics of the dataset, PSM offers a data-driven approach to bias adjustment that balances class distributions and demographic variables without introducing significant model complexity. Therefore, it can be a useful option in practical applications where selection bias is a concern, such as when working with real-world data.

### 4.4 Limitations and future directions

This study quantitatively analyzes the relationship between selection bias and model performance. Moreover, unlike prior studies focusing primarily on statistical balance or treatment effect estimation, our work highlights how PSM can influence downstream machine learning classification performance when applied to observational medical datasets. However, it has certain limitations. First, all experiments were conducted on publicly available datasets, which may not fully reflect the complexity of clinical data from diverse institutions. In particular, the datasets used in this study were not considered to be fully representative of the general population, but rather were interpreted as reflecting their respective data-generating processes and clinical care contexts. Second, whereas our study primarily focuses on selection bias, further validation is needed across datasets of varying sizes. Additionally, we compare only 1:1 and 1:4 matching ratios in PSM. Future research should investigate the effect of using dataset-specific optimal matching ratios. Such efforts would lead to more tailored and effective strategies for selection bias adjustment across diverse real-world medical datasets. Lastly, while our study emphasizes statistical bias reduction, additional evaluation of clinical relevance, such as impact on diagnostic decision-making or subgroup fairness, would enhance the practical significance of the findings and help support their generalizability and utility in real-world healthcare settings.

## 5 Conclusions

This study analyzes the impact of PSM as a method for correcting selection bias and assessing its influence on classification performance in machine learning–based models. To achieve this, we compare PSM with various resampling techniques across multiple medical datasets (RPD, HypoT, and CVD) and quantitatively assess the effect of selection bias on data imbalance and model performance.

The results demonstrate that PSM effectively reduces the SMD and maintains stable classification performance when applied to datasets containing demographic variables with low selection bias. Additionally, PSM contributes to enhancing the internal validity of models. However, when applied to datasets with highly biased variables, or when overly restrictive matching is employed, PSM results in a decline in model performance. This finding highlights the necessity of carefully considering dataset characteristics and matching intensity when employing PSM.

These findings emphasize the importance of selecting appropriate resampling techniques when utilizing machine learning in real-world medical research settings. Although this study does not explore the influence of the size of the dataset or the determination of optimal matching ratios, future work may address these limitations by incorporating a broader range of medical datasets and systematically evaluating alternative matching configurations.

## Data Availability

Restrictions apply to the availability of RPD data. Data were obtained from the Alzheimer's Disease Neuroimaging Initiative (ADNI) and are available at adni.loni.usc.edu with the permission of ADNI. The original HypoT data presented in the study are openly available in UCI Machine Learning Repository at https://doi.org/10.24432/C5D010. The original CVD data presented in the study are openly available in Kaggle at https://www.kaggle.com/datasets/soumyodippal000/heart-disease-health-indicators. This work conforms to the TRIPOD-AI guidelines (31), with the completed checklist included in the Supplementary material S7. The code used for this study is publicly available at https://github.com/knu-plml/psm-medical-ml-bias.
