# Supplementary material for: Evaluating the sampling effect of propensity score matching for reducing selection bias in medical data
Source: Front Public Health. 2026 Feb 10;14:1747762. doi: 10.3389/fpubh.2026.1747762 (PMC12929392; doi:10.3389/fpubh.2026.1747762)
Supplement: Supplementary file 1 [file Data_Sheet_1.docx]

Table of Contents (Supplementary Materials)

[Supplementary Material S1. Baseline Characteristics of the Study Datasets 2](#_Toc217552400)

[Supplementary Material S2. Settings for Classification Models 6](#_Toc217552401)

[Supplementary Material S3. Evaluation Metrics 7](#_Toc217552402)

[Supplementary Material S4. Model Selection Based on AUPRC 10](#_Toc217552403)

[Supplementary Material S5. Distributional Characteristics of Resampled Datasets 11](#_Toc217552404)

[Supplementary Material S6. Detailed Performance Metrics Associated with the Main Results 12](#_Toc217552405)

[Supplementary Material S7. TRIPOD+AI Checklist 24](#_Toc217552406)

# Supplementary Material S1. Baseline Characteristics of the Study Datasets

**Supplementary Table S1. Baseline Characteristics of the ADNI Cohort used for RPD Construction**

| **Variables** | **Screening** | **6 months** | | **12 months** | | **24 months** | | **36 months** | |
| --- | --- | --- | --- | --- | --- | --- | --- | --- | --- |
| **N (Baseline)** | 790 | | | | | | | | |
| **Age (years)** | 73.44±7.07 | | | | | | | | |
| **Sex** |  | | | | | | | | |
| Male | 458 (57.97%) | | | | | | | | |
| Female | 332 (42.03%) | | | | | | | | |
| **Education (years)** | 16.07±2.77 | | | | | | | | |
| **APOE4** |  | | | | | | | | |
| 0 | 442 (55.95%) | | | | | | | | |
| 1 | 269 (34.05%) | | | | | | | | |
| 2 | 79 (10.00%) | | | | | | | | |
| **CDRSB** | | | | | | | | | |
| N | 790 | | 783 | | 781 | | 774 | | 757 |
| Mean±SD | 1.19±1.13 | | 1.36±1.43 | | 1.49±1.62 | | 1.93±2.27 | | 2.36±3.00 |
| **FAQ** | | | | | | | | | |
| N | 787 | | 786 | | 783 | | 778 | | 765 |
| Mean±SD | 2.62±4.26 | | 3.14±4.80 | | 3.75±5.72 | | 4.89±7.06 | | 6.1±8.39 |
| **MMSE** | | | | | | | | | |
| N | 790 | | 790 | | 790 | | 778 | | 761 |
| Mean±SD | 27.92±1.97 | | 27.61±2.44 | | 27.55±2.84 | | 27.03±3.56 | | 26.53±4.35 |
| **Hippocampus** | | | | | | | | | |
| N | 685 | | 660 | | 650 | | 619 | | 353 |
| Mean±SD | 6950.08±1131.39 | | 6878.26±1155.5 | | 6826.90±1186.74 | | 6730.70±1237.21 | | 6653.93±1231.28 |
| **Entorhinal Cortex** | | | | | | | | | |
| N | 672 | | 643 | | 627 | | 555 | | 333 |
| Mean±SD | 3593.77±762.97 | | 3554.65±806.73 | | 3530.08±786.48 | | 3467.65±813.47 | | 3435.80±811.15 |
| **Fusiform Gyrus** | | | | | | | | | |
| N | 672 | | 643 | | 627 | | 555 | | 333 |
| Mean±SD | 17593.61±2672.11 | | 17454.38±2700.83 | | 17391.68±2751.44 | | 17213.15±2860.87 | | 16649.40±2742.41 |
| **Middle Temporal Gyrus** | | | | | | | | | |
| N | 672 | | 643 | | 627 | | 555 | | 333 |
| Mean±SD | 19812.40±2827.59 | | 19615.12±2891.23 | | 19474.26±2887.75 | | 19270.64±2978.18 | | 18769.82±3097.98 |
| Definitions: CDRSB (Clinical Dementia Rating Sum of Boxes), FAQ (Functional Activities Questionnaire), and MMSE (Mini Mental State Examination) are cognitive assessment scores. MRI-derived variables (hippocampus, entorhinal cortex, fusiform gyrus, and middle temporal gyrus) represent region-of-interest–averaged continuous measures expressed in arbitrary units (a.u.). All variables are summarized as mean ± standard deviation. | | | | | | | | | |

**Supplementary Table S2. Baseline Characteristics of the RPD Dataset (Before Resampling and Propensity Score Matching)**

| **Variables** | **Patients with Slowly Progressive Dementia (SPD)** | **Patients with Rapidly Progressive Dementia**  **(RPD)** | **P-Value** | **SMD** |
| --- | --- | --- | --- | --- |
| **N** | 51 | 577 |  |  |
| **Age (years)** | 73.76±6.31 | 73.09±6.92 | 0.508 | 0.100 |
| **Sex** |  |  | 0.828 | 0.053 |
| Male | 28 (54.9%) | 332 (57.5%) |  |  |
| Female | 23 (45.1%) | 245 (42.5%) |  |  |
| **Education (years)** | 15.69±3.33 | 16.10±2.77 | 0.316 | 0.135 |
| **APOE4** |  |  | <0.001*** | 0.682 |
| 0 | 15 (29.4%) | 341 (59.1%) |  |  |
| 1 | 23 (45.1%) | 189 (32.8%) |  |  |
| 2 | 13 (25.5%) | 47 (8.1%) |  |  |
| **FAQ** | 8.10±6.80 | 2.03±3.53 | <0.001*** | 1.121 |
| **MMSE** | 25.80±2.34 | 28.19±1.71 | <0.001*** | 1.162 |
| **Hippocampus** | 5828.10±940.83 | 7056.73±1080.17 | <0.001*** | 1.213 |
| **Entorhinal Cortex** | 2810.41±708.03 | 3655.21±727.88 | <0.001*** | 1.177 |
| **Fusiform Gyrus** | 15877.75±2357.96 | 17670.88±2533.30 | <0.001*** | 0.733 |
| **Middle Temporal Gyrus** | 17323.75±2843.43 | 19970.64±2698.87 | <0.001*** | 0.955 |
| * p < 0.05, ∗∗p < 0.01, ∗∗∗p < 0.001  Definitions: Cognitive assessment scores (CDRSB, FAQ, MMSE) are reported as total scores. MRI-derived variables are expressed in arbitrary units (a.u.). | | | | |

**Supplementary Table S3. Baseline Characteristics of the HypoT Dataset**

| **Variables** | **Healthy Controls** | **Patients with Hypothyroidism** | **P-Value** | **SMD** |
| --- | --- | --- | --- | --- |
| **N** | 291 | 3,481 |  |  |
| **Age (years)** | 51.52±19.11 | 51.76±20.17 | 0.842 | 0.012 |
| **Sex** |  |  | 0.003** | 0.196 |
| Male | 65 (22.3%) | 1,077 (30.9%) |  |  |
| Female | 226 (77.7%) | 2,404 (69.1%) |  |  |
| **TSH (μIU/mL)** | 2.17±10.21 | 39.23±74.38 | <0.001*** | 0.698 |

* p < 0.05, ∗∗p < 0.01, ∗∗∗p < 0.001; TSH: Thyroid-Stimulating Hormone (μIU/mL)

**Supplementary Table S4. Baseline Characteristics of the CVD Dataset**

| **Variables** | **Healthy Controls** | **Patients with Cardiovascular Disease** | **P-Value** | **SMD** |
| --- | --- | --- | --- | --- |
| **N** | 229,787 | 23,893 |  |  |
| **Age (5-year groups)** |  |  | <0.001*** | 0.334 |
| 18-24 | 5,671 (2.5%) | 29 (0.1%) |  |  |
| 25-29 | 7,544 (3.3%) | 54 (0.2%) |  |  |
| 30-34 | 10,997 (4.8%) | 126 (0.5%) |  |  |
| 35-39 | 13,630 (5.9%) | 193 (0.8%) |  |  |
| 40-44 | 15,806 (6.9%) | 351 (1.5%) |  |  |
| 45-49 | 19,107 (8.3%) | 712 (3.0%) |  |  |
| 50-54 | 24,889 (10.8%) | 1,425 (6.0%) |  |  |
| 55-59 | 28,579 (12.4%) | 2,253 (9.4%) |  |  |
| 60-64 | 29,886 (13.0%) | 3,358 (14.1%) |  |  |
| 65-69 | 28,001 (12.2%) | 4,193 (17.5%) |  |  |
| 70-74 | 19,586 (8.5%) | 3,947 (16.5%) |  |  |
| 75-79 | 12,887 (5.6%) | 3,093 (12.9%) |  |  |
| 80+ | 13,204 (5.7%) | 4,159 (17.4%) |  |  |
| **Sex** |  |  | <0.001*** | 0.296 |
| Male | 131,769 (57.3%) | 10,205 (42.7%) |  |  |
| Female | 98,018 (42.7%) | 13,688 (57.3%) |  |  |
| **Education** |  |  | <0.001*** | 0.140 |
| Less than elementary school | 145 (0.1%) | 29 (0.1%) |  |  |
| Elementary school graduate | 3,265 (1.4%) | 778 (3.3%) |  |  |
| Middle school graduate | 7,860 (3.4%) | 1,618 (6.8%) |  |  |
| High school graduate | 55,283 (24.1%) | 7,467 (31.3%) |  |  |
| Some college or technical school | 62,992 (27.4%) | 6,918 (29.0%) |  |  |
| College or technical school graduate | 100,242 (43.6%) | 7,083 (29.6%) |  |  |
| **Income** |  |  | <0.001*** | 0.212 |
| <$10,000 | 8,258 (3.6%) | 1,553 (6.5%) |  |  |
| $10,000-<$15,000 | 9,586 (4.2%) | 2,197 (9.2%) |  |  |
| $15,000-<$20,000 | 13,475 (5.9%) | 2,519 (10.5%) |  |  |
| $20,000-<$25,000 | 17,307 (7.5%) | 2,828 (11.8%) |  |  |
| $25,000-<$35,000 | 22,722 (9.9%) | 3,161 (13.2%) |  |  |
| $35,000-<$50,000 | 32,824 (14.3%) | 3,646 (15.3%) |  |  |
| $50,000-<$75,000 | 39,815 (17.3%) | 3,404 (14.2%) |  |  |
| ≥$75,000 | 85,800 (37.3%) | 4,585 (19.2%) |  |  |
| **Health Insurance Coverage** |  |  | <0.001*** | 0.014 |
| No | 11,547 (5.0%) | 870 (3.6%) |  |  |
| Yes | 218,240 (95.0%) | 23,023 (96.4%) |  |  |
| **BMI** | 28.27±6.58 | 29.47±6.74 | <0.001*** | 0.181 |
| **Currently Smoking** |  |  | <0.001*** | 0.195 |
| No | 132,165 (57.5%) | 9,092 (38.1%) |  |  |
| Yes | 97,622 (42.5%) | 14,801 (61.9%) |  |  |
| **Heavy Alcohol Consumption** |  |  | <0.001*** | 0.023 |
| No | 216,379 (94.2%) | 23,045 (96.5%) |  |  |
| Yes | 13,408 (5.8%) | 848 (3.5%) |  |  |
| **Hypertension Diagnosis** |  |  | <0.001*** | 0.355 |
| No | 138,886 (60.4%) | 5,965 (25.0%) |  |  |
| Yes | 90,901 (39.6%) | 17,928 ( 75.0%) |  |  |
| **High Cholesterol Diagnosis** |  |  | <0.001*** | 0.306 |
| No | 138,949 (60.5%) | 7,140 (29.9%) |  |  |
| Yes | 90,838 (39.5%) | 16,753 (70.1%) |  |  |
| **Stroke Diagnosis** |  |  | <0.001*** | 0.137 |
| No | 223,432 (97.2%) | 19,956 (83.5%) |  |  |
| Yes | 6,355 (2.8%) | 3,937 (16.5%) |  |  |
| **Diabetes Diagnosis** |  |  | <0.001*** | 0.221 |
| No diabetes | 198,352 (86.3%) | 15,351 (64.2%) |  |  |
| Pre-diabetes | 3,967 (1.7%) | 664 (2.8%) |  |  |
| Diabetes | 27,468 (12.0%) | 7,878 (33.0%) |  |  |
| **Cholesterol Screening (Past 5 Years)** |  |  | <0.001*** | 0.029 |
| No | 9,199 (4.0%) | 271 (1.1%) |  |  |
| Yes | 220,588 (96.0%) | 23,622 (98.9%) |  |  |
| **Physical Activity (Past 30 Days)** |  |  | <0.001*** | 0.128 |
| No | 53,167 (23.1%) | 8,593 (36.0%) |  |  |
| Yes | 176,620 (76.9%) | 15,300 (64.0%) |  |  |
| **Daily Fruit Consumption** |  |  | <0.001*** | 0.033 |
| No | 83,337 (36.3%) | 9,445 (39.5%) |  |  |
| Yes | 146,450 (63.7%) | 14,448 (60.5%) |  |  |
| **Daily Vegetable Consumption** |  |  | <0.001*** | 0.052 |
| No | 42,198 (18.4%) | 5,641 (23.6%) |  |  |
| Yes | 187,589 (81.6%) | 18,252 (76.4%) |  |  |
| **Cost-Related Barriers to Care  (Past 12 Months)** |  |  | <0.001*** | 0.029 |
| No | 211,082 (91.9%) | 21,244 (88.9%) |  |  |
| Yes | 18,705 (8.1%) | 2,649 (11.1%) |  |  |
| **Self-Rated General Health** |  |  | <0.001*** | 0.347 |
| 1 | 44,283 (19.3%) | 1,016 (4.3%) |  |  |
| 2 | 84,956 (37.0%) | 4,128 (17.3%) |  |  |
| 3 | 67,732 (29.5%) | 7,914 (33.1%) |  |  |
| 4 | 24,842 (10.8%) | 6,728 (28.2%) |  |  |
| 5 | 7,974 (3.5%) | 4,107 (17.2%) |  |  |
| **Mental Health (Poor Health Days)** | 3.03±7.18 | 4.67±9.19 | <0.001*** | 0.222 |
| **Physical Health (Poor Health Days)** | 3.73±8.15 | 9.15±11.87 | <0.001*** | 0.633 |
| **Serious Difficulty Walking**  **or Climbing Stairs** |  |  | <0.001*** | 0.272 |
| No | 197,027 (85.7%) | 13,978 (58.5%) |  |  |
| Yes | 32,760 (14.3%) | 9,915 (41.5%) |  |  |
| * p < 0.05, ∗∗p < 0.01, ∗∗∗p < 0.001; Abbreviations and definitions: Health insurance coverage includes health insurance, HMO, or other healthcare plans; heavy alcohol consumption was defined as ≥14 drinks per week for men or ≥7 drinks per week for women; mental health and physical health represent the number of days with poor health in the past 30 days. | | | | |

# Supplementary Material S2. Settings for Classification Models

**Supplementary Table S5. Hyperparameter Settings for Machine Learning Classification Models**

| **Models** | **Hyperparameter Settings** |
| --- | --- |
| **Logistic Regression** | random_state=random_state, max_iter=1000, C=1.0, dual=False, tol=0.0001, fit_intercept=True, intercept_scaling=1, class_weight=None, solver='lbfgs', verbose=0, warm_start=False, n_jobs=None |
| **Random Forest** | random_state=random_state, n_estimators=100, criterion='gini', max_depth=None, min_samples_split=2, min_samples_leaf=1, min_weight_fraction_leaf=0.0, max_features='sqrt', max_leaf_nodes=None, min_impurity_decrease=0.0, bootstrap=True, oob_score=False, n_jobs=None, verbose=0, warm_start=False, class_weight=None, ccp_alpha=0.0, max_samples=None, monotonic_cst=None |
| **XGBoost** | random_state=random_state, eval_metric='logloss', objective='binary:logistic' |
| **LightGBM** | random_state=random_state, verbose=-1, boosting_type='gbdt', num_leaves=31, max_depth=-1, learning_rate=0.1, n_estimators=100, subsample_for_bin=200000, objective=None, class_weight=None, min_split_gain=0.0, min_child_weight=0.001, min_child_samples=20, subsample=1.0, subsampler=0, colsample_bytree=1.0, reg_alpha=0.0, reg_lambda=0.0, n_jobs=None, importance_type='split' |
| **SVM** | kernel='rbf', probability=True, random_state=random_state, C=1.0, degree=3, gamma='scale', coef0=0.0, shrinking=True, tol=0.001, cache_size=200, class_weight=None, verbose=False, max_iter=-1, decision_function_shape='ovr', break_ties=False |
| **Naïve Bayes** | priors=None, var_smoothing=1e-9 |
| Note: All classification models were trained using fixed hyperparameter settings without tuning to ensure fair comparison across resampling strategies. Default parameters were used unless otherwise specified. Random seeds were controlled for reproducibility. | |

# Supplementary Material S3. Evaluation Metrics

All classification models produced probabilistic predictions $\hat{p}(y=1|x)$. For threshold-dependent metrics, such as accuracy, precision, recall, F1 score, and specificity, predicted probabilities were converted into binary class labels using a fixed probability threshold of 0.5. This threshold represents the standard default in binary classification and is widely adopted in medical AI studies when domain-specific risk thresholds have not been established[S1-S4].

Threshold-independent evaluations, such as Receiver Operating Characteristic (ROC) curves, Precision-Recall (PR) curves, calibration curves, Brier Score and Decision Curve Analysis(DCA) , utilized the complete distribution of predicted probabilities, thereby avoiding dependence on any fixed classification threshold.

1. **Threshold-Dependent Classification Metrics**

Let TP, TN, FP, and FN denote the numbers of true positives, true negatives, false positives, and false negatives, respectively, in the confusion matrix.

Supplementary Table S6. Confusion Matrix

|  | | **Predicted Class** | |
| --- | --- | --- | --- |
|  |  | **Positive** | **Negative** |
| **Actual**  **Class** | **Positive** | TP | FN |
|  | **Negative** | FP | TN |

The following metrics were calculated:

$$Accuracy= \frac{TP+TN}{TP+TN+FP+FN}$$

$$Precision= \frac{TP}{TP+FP}$$

$$Recall\left( Sensitivity \right)= \frac{TP}{TP+FN}$$

$$Specificity= \frac{TN}{TN+FP}$$

$$F1 Score=\frac{2\times Precision\times Recall}{Precision+Recall}$$

These metrics were computed after converting predicted probabilities into binary labels using a probability threshold of 0.5.

**2. Threshold-Independent Discrimination Metrics**

2-1. ROC Curve and AUROC

The ROC curve plots the true positive rate against the false positive rate across all possible probability thresholds. The area under the ROC curve (AUROC) summarizes the model’s overall discrimination ability, independent of any specific classification threshold.

2-2. Precision–Recall Curve and AUPRC

The precision–recall (PR) curve depicts the trade-off between precision and recall over varying decision thresholds. The area under the PR curve (AUPRC) is particularly informative for imbalanced datasets, where it more accurately reflects performance on the positive class.

2-3. Calibration Curve

Model calibration was evaluated using calibration curves, which compare predicted probabilities with observed outcome frequencies. Predicted probabilities were partitioned into bins of equally sized bins, and the mean predicted probability within each bin was plotted against the corresponding observed frequency. Well-calibrated models exhibit predictions that closely follow the diagonal line representing perfect calibration.

2-4. Brier Score

Model calibration was additionally quantified using the Brier score, which measures the mean squared difference between predicted probabilities and observed binary outcomes. The Brier score is defined as:

$$Brier score= \frac{1}{N}\sum_{i=1}^{N} {(\hat{p}_{i}-y_{i})}^{2}$$

where $\hat{p}_{i}$ denotes the predicted probability for sample $i$, $y_{i}\in\{0,1\}$is the true outcome, and $N$is the total number of samples. Lower Brier scores indicate better probabilistic accuracy, reflecting both discrimination and calibration performance. Unlike threshold-dependent classification metrics, the Brier score was computed directly from predicted probabilities without applying a fixed classification threshold.

2-5. Decision Curve Analysis

Clinical utility was evaluated using decision curve analysis (DCA), which assesses the net benefit of a prediction model across a range of decision thresholds $p_{t}$. Net benefit was calculated as:

$$Net Benefit= \frac{TP}{N}- \frac{FP}{N}\times\frac{p_{t}}{1-p_{t}}$$

where $N$ denotes the total number of samples. DCA curves were compared against default strategies of treating all patients and treating none, allowing assessment of whether the model provides incremental clinical value across plausible risk thresholds.

**References**

[S1] PEDREGOSA, Fabian, et al. Scikit-learn: Machine learning in Python. *the Journal of machine Learning research*, 2011, 12: 2825-2830.

[S2] scikit-learn developers. Model selection and evaluation. scikit-learn documentation. https://scikit-learn.org/stable/modules/model_evaluation.html (accessed 2025).

[S3] PARK, Seong Ho, et al. Methods for clinical evaluation of artificial intelligence algorithms for medical diagnosis. Radiology, 2023, 306.1: 20-31.

[S4] KLONTZAS, Michail E., et al. ESR Essentials: common performance metrics in AI—practice recommendations by the European Society of Medical Imaging Informatics. European Radiology, 2025, 1-13.

# Supplementary Material S4. Model Selection Based on AUPRC

**Supplementary Table S7. Mean AUPRC of Each Model**

| **Dataset**  **Model** | **RPD** | **HypoT** | **CVD** |
| --- | --- | --- | --- |
| **Logistic Regression** | **0.5238±0.0733** | 0.8583±0.3400 | **0.3249±0.1103** |
| **Random Forest** | 0.4431±0.1337 | 0.8591±0.3389 | 0.2692±0.0900 |
| **XGBoost** | 0.4315±0.1225 | **0.8594±0.3402** | 0.3154±0.1065 |
| **LGBM** | 0.4334±0.1095 | 0.8592±0.3402 | 0.3247±0.1102 |
| **Naïve Bayes** | 0.5130±0.0976 | 0.8583±0.3387 | 0.2707±0.0880 |
| **Soft Voting** | 0.4747±0.1176 | 0.8590±0.3403 | 0.3192±0.1090 |

This table summarizes the mean Area Under the Precision–Recall Curve (AUPRC) for each classification model across all resampling strategies in the RPD, HypoT, and CVD datasets. Reported values represent the mean ± standard deviation of out-of-fold AUPRC obtained from 10 repetitions of 5-fold cross-validation.

To identify a representative model for each dataset, model selection was performed using a pre-defined criterion based on the average AUPRC across all resampling methods. This approach was adopted to ensure robust discrimination performance under class imbalance while minimizing the influence of stochastic variation introduced by resampling and cross-validation. The selected models served as fixed reference models for subsequent analyses comparing the effects of different resampling and propensity score matching strategies, rather than for further performance optimization. Based on this criterion, logistic regression was selected as the representative model for the RPD dataset, XGBoost for the HypoT dataset, and logistic regression for the CVD dataset.

# Supplementary Material S5. Distributional Characteristics of Resampled Datasets

**Supplementary Table S8. Class Distribution and Imbalance Statistics across Resampling Techniques**

|  |  |  | (Mean ± SD) |
| --- | --- | --- | --- |
| **Resampling Techniques** | **Healthy Samples** | **Patient Samples** | **Imbalance Ratio** |
| **RPD Dataset** | | | |
| Baseline | 461.60±0.49 | 40.80±0.40 | 11.31±0.12 |
| RUS | 40.80±0.40 | 40.80±0.40 | 1.00±0.00 |
| TL | 444.94±2.76 | 40.80±0.40 | 10.91±0.14 |
| OSS | 416.76±23.60 | 40.80±0.40 | 10.22±0.58 |
| ENN | 377.26±5.81 | 40.80±0.40 | 9.25±0.18 |
| NCR | 384.24±5.08 | 40.80±0.40 | 9.42±0.17 |
| ROS | 461.60±0.49 | 461.60±0.49 | 1.00±0.00 |
| SMOTE | 461.60±0.49 | 461.60±0.49 | 1.00±0.00 |
| ADASYN | 461.60±0.49 | 461.56±7.03 | 1.01±0.01 |
| SMOTE_TL | 449.62±2.70 | 449.62±2.70 | 1.00±0.00 |
| SMOTE_ENN | 300.60±17.79 | 398.22±10.56 | 1.33±0.06 |
| OUPS | 461.60±0.49 | 461.60±0.49 | 1.00±0.00 |
| PSM (1:4) | 163.20±1.60 | 40.80±0.40 | 4.00±0.00 |
| PSM (1:1) | 40.80±0.40 | 40.80±0.40 | 1.00±0.00 |
| **HypoT Dataset** | | | |
| Baseline | 232.80±0.40 | 2784.80±0.40 | 11.96±0.02 |
| RUS | 232.80±0.40 | 232.80±0.40 | 1.00±0.00 |
| TL | 232.80±0.40 | 2745.24±4.06 | 11.79±0.03 |
| OSS | 232.80±0.40 | 2416.72±144.81 | 10.38±0.62 |
| ENN | 232.80±0.40 | 2661.36±6.33 | 11.43±0.03 |
| NCR | 232.80±0.40 | 2673.96±6.75 | 11.49±0.03 |
| ROS | 2784.80±0.40 | 2784.80±0.40 | 1.00±0.00 |
| SMOTE | 2784.80±0.40 | 2784.80±0.40 | 1.00±0.00 |
| ADASYN | 2781.34±28.55 | 2784.80±0.40 | 1.01±0.01 |
| SMOTE_TL | 2762.22±4.50 | 2762.22±4.50 | 1.00±0.00 |
| SMOTE_ENN | 2659.08±13.57 | 2638.72±11.18 | 1.01±0.01 |
| OUPS | 2784.80±0.40 | 2784.80±0.40 | 1.00±0.00 |
| PSM (1:4) | 232.80±0.40 | 931.20±1.60 | 4.00±0.00 |
| PSM (1:1) | 232.80±0.40 | 232.80±0.40 | 1.00±0.00 |
| **CVD Dataset** | | | |
| Baseline | 183829.60±0.49 | 19114.40±0.49 | 9.62±0.00 |
| RUS | 19114.40±0.49 | 19114.40±0.49 | 1.00±0.00 |
| TL | 178897.42±44.17 | 19114.40±0.49 | 9.36±0.00 |
| OSS | 179850.50±90.40 | 19114.40±0.49 | 9.41±0.00 |
| ENN | 149510.80±122.90 | 19114.40±0.49 | 7.82±0.01 |
| NCR | 153538.80±100.02 | 19114.40±0.49 | 8.03±0.01 |
| ROS | 183829.60±0.49 | 183829.60±0.49 | 1.00±0.00 |
| SMOTE | 183829.60±0.49 | 183829.60±0.49 | 1.00±0.00 |
| ADASYN | 183829.60±0.49 | 185129.02±273.70 | 1.01±0.00 |
| SMOTE_TL | 183763.04±7.23 | 183763.04±7.23 | 1.00±0.00 |
| SMOTE_ENN | 123948.88±173.91 | 178273.44±95.38 | 1.44±0.00 |
| OUPS | 183829.60±0.49 | 183829.60±0.49 | 1.00±0.00 |
| PSM (1:4) | 76457.60±1.96 | 19114.40±0.49 | 4.00±0.00 |
| PSM (1:1) | 19114.40±0.49 | 19114.40±0.49 | 1.00±0.00 |

# Supplementary Material S6. Detailed Performance Metrics Associated with the Main Results

Supplementary Material S6 provides detailed numerical summaries (mean ± SD) and additional visualizations corresponding to the results presented in the main figures. Supplementary Tables S9–S11 report classification performance metrics computed across 10 random seeds with 5-fold cross-validation for each resampling and PSM strategy, under conditions including and excluding demographic variables. These materials support the comparative analyses discussed in the Results and Discussion sections. In Supplementary Tables S9–S11, the best-performing result for each metric across resampling strategies is highlighted in bold.

**Supplementary Table S9. Classification Performance Metrics for the RPD Dataset With and Without Demographic Variables**

|  |  |  |  |  |  |  |  | (Mean ± SD) |
| --- | --- | --- | --- | --- | --- | --- | --- | --- |
| **Resampling Techniques** | **Accuracy** | **Precision** | **Recall** | **F1 Score** | **Specificity** | **AUROC** | **AUPRC** | **Brier Score** |
| Including demographic variables | | | | | | | | |
| Baseline | 0.9218±0.0057 | 0.3976±0.0857 | 0.2400±0.1148 | 0.2907±0.1071 | **0.9736±0.0095** | 0.9054±0.0401 | 0.4846±0.0566 | 0.0521±0.0038 |
| RUS | 0.8288±0.0367 | 0.2699±0.0415 | 0.8142±0.1966 | 0.4013±0.0646 | 0.8296±0.0477 | 0.9129±0.0414 | 0.4782±0.1124 | 0.1261±0.0250 |
| TL | 0.9241±0.0067 | 0.4327±0.1019 | 0.3304±0.1238 | 0.3712±0.1197 | 0.9693±0.0048 | 0.9062±0.0371 | 0.4729±0.0576 | 0.0530±0.0043 |
| OSS | 0.9234±0.0074 | 0.4336±0.0918 | 0.3171±0.1186 | 0.3600±0.1104 | 0.9696±0.0085 | 0.9072±0.0375 | 0.4713±0.0645 | 0.0530±0.0046 |
| ENN | 0.9177±0.0096 | 0.4201±0.0795 | 0.4689±0.1310 | 0.4403±0.1019 | 0.9520±0.0080 | 0.9001±0.0400 | 0.4622±0.0943 | 0.0578±0.0048 |
| NCR | **0.9243±0.0136** | 0.4618±0.1182 | 0.4556±0.1603 | 0.4520±0.1288 | 0.9601±0.0123 | 0.9037±0.0394 | 0.4662±0.0999 | 0.0551±0.0073 |
| ROS | 0.9022±0.0222 | 0.3695±0.1072 | 0.4464±0.1337 | 0.3917±0.0834 | 0.9367±0.0284 | 0.9108±0.0339 | 0.4396±0.0848 | 0.0661±0.0115 |
| SMOTE | 0.8964±0.0246 | 0.3422±0.1206 | 0.4638±0.1703 | 0.3857±0.1231 | 0.9294±0.0264 | 0.9079±0.0369 | 0.4212±0.0976 | 0.0719±0.0125 |
| ADASYN | 0.8960±0.0233 | 0.3377±0.1177 | 0.4596±0.1687 | 0.3822±0.1221 | 0.9292±0.0241 | 0.9082±0.0376 | 0.4273±0.1052 | 0.0726±0.0131 |
| SMOTE_TL | 0.8963±0.0247 | 0.3442±0.1176 | 0.4831±0.1890 | 0.3926±0.1262 | 0.9277±0.0279 | 0.9071±0.0364 | 0.4193±0.1006 | 0.0723±0.0123 |
| SMOTE_ENN | 0.8844±0.0224 | 0.3488±0.0621 | 0.7178±0.1881 | 0.4643±0.0880 | 0.8970±0.0286 | 0.8995±0.0364 | 0.3896±0.1026 | 0.095±0.0194 |
| OUPS | 0.8833±0.0292 | 0.3304±0.1141 | 0.6000±0.2175 | 0.4191±0.1338 | 0.9046±0.0321 | 0.9055±0.0358 | 0.4513±0.0942 | 0.0813±0.014 |
| PSM (1:4) | 0.9239±0.0178 | **0.4869±0.0777** | 0.7916±0.1059 | **0.5984±0.0718** | 0.9339±0.0191 | **0.9529±0.0262** | **0.6872±0.1083** | 0.0558±0.0108 |
| PSM (1:1) | 0.7948±0.0361 | 0.2510±0.0321 | **0.9251±0.082** | 0.3934±0.0413 | 0.7848±0.0413 | 0.9274±0.0253 | 0.5743±0.1107 | **0.1485±0.0191** |
| Excluding demographic variables | | | | | | | | |
| Baseline | **0.9301±0.0093** | **0.4902±0.1266** | 0.3178±0.1485 | 0.3762±0.1460 | **0.9766±0.0084** | 0.9116±0.0313 | 0.4977±0.0754 | 0.0510±0.0041 |
| RUS | 0.8323±0.0355 | 0.2750±0.0426 | **0.8162±0.1955** | 0.4072±0.0642 | 0.8333±0.0466 | **0.9193±0.0378** | 0.4743±0.1345 | **0.1239±0.0245** |
| TL | 0.9273±0.0127 | 0.4549±0.1451 | 0.3422±0.1659 | 0.3855±0.1623 | 0.9717±0.0072 | 0.9087±0.0330 | **0.5002±0.0801** | 0.0527±0.0049 |
| OSS | 0.9273±0.0121 | 0.4650±0.1315 | 0.3533±0.1530 | 0.3958±0.1452 | 0.9709±0.0085 | 0.9096±0.0346 | 0.4878±0.0845 | 0.0524±0.0051 |
| ENN | 0.9171±0.0080 | 0.4153±0.0725 | 0.4889±0.1502 | 0.4459±0.1079 | 0.9498±0.0056 | 0.9054±0.0353 | 0.4803±0.1125 | 0.0579±0.0060 |
| NCR | 0.9231±0.0157 | 0.4689±0.1137 | 0.4778±0.1309 | **0.4664±0.1042** | 0.9571±0.0158 | 0.9046±0.0414 | 0.4851±0.1252 | 0.0556±0.0077 |
| ROS | 0.9034±0.0263 | 0.3891±0.1146 | 0.5440±0.1759 | 0.4428±0.1166 | 0.9307±0.0300 | 0.9130±0.0273 | 0.4327±0.0611 | 0.0688±0.0118 |
| SMOTE | 0.9004±0.0291 | 0.3778±0.1387 | 0.5780±0.2291 | 0.4474±0.1538 | 0.9251±0.0297 | 0.9172±0.0301 | 0.4395±0.1295 | 0.0746±0.0156 |
| ADASYN | 0.8987±0.0300 | 0.3728±0.1436 | 0.5784±0.2271 | 0.4451±0.1578 | 0.9232±0.0287 | 0.9169±0.0319 | 0.4436±0.1460 | 0.0752±0.0164 |
| SMOTE_TL | 0.8980±0.0281 | 0.3680±0.1292 | 0.5711±0.2149 | 0.4393±0.1441 | 0.9229±0.0285 | 0.9162±0.0304 | 0.4384±0.1276 | 0.0749±0.0158 |
| SMOTE_ENN | 0.8803±0.0177 | 0.3414±0.0431 | 0.7360±0.1640 | 0.4624±0.0639 | 0.8911±0.0264 | 0.9068±0.0310 | 0.4180±0.1194 | 0.1005±0.0212 |
| OUPS | 0.8875±0.0270 | 0.3539±0.1004 | 0.6793±0.2104 | 0.4587±0.1204 | 0.9031±0.0313 | 0.9181±0.0299 | 0.4713±0.1353 | 0.0825±0.0147 |

**Supplementary Table S10. Classification Performance Metrics for the HypoT Dataset With and Without Demographic Variables**

|  |  |  |  |  |  |  |  | (Mean ± SD) |
| --- | --- | --- | --- | --- | --- | --- | --- | --- |
| **Resampling Techniques** | **Accuracy** | **Precision** | **Recall** | **F1 Score** | **Specificity** | **AUROC** | **AUPRC** | **Brier Score** |
| Including demographic variables | | | | | | | | |
| Baseline | 0.9717±0.0049 | 0.9813±0.0053 | **0.9893±0.0051** | 0.9852±0.0026 | 0.6217±0.1114 | 0.9647±0.0273 | 0.9971±0.003 | 0.0234±0.0041 |
| RUS | 0.9486±0.0169 | 0.9934±0.0055 | 0.9524±0.0217 | 0.9723±0.0094 | 0.8714±0.1109 | 0.9693±0.0213 | 0.9977±0.0026 | 0.0518±0.0118 |
| TL | 0.9722±0.0055 | 0.9857±0.0053 | 0.9852±0.0074 | 0.9854±0.0029 | 0.7131±0.1099 | 0.9679±0.0268 | 0.9974±0.0028 | 0.0233±0.0052 |
| OSS | **0.9723±0.0053** | 0.9855±0.0055 | 0.9855±0.0075 | **0.9855±0.0028** | 0.7085±0.1136 | 0.9693±0.0245 | 0.9976±0.0026 | 0.0232±0.0050 |
| ENN | 0.9647±0.0076 | 0.9908±0.0043 | 0.9720±0.0093 | 0.9813±0.0041 | 0.8178±0.0870 | 0.9708±0.0234 | 0.9976±0.0026 | 0.0299±0.0077 |
| NCR | 0.9669±0.0079 | 0.9897±0.0050 | 0.9754±0.0088 | 0.9825±0.0042 | 0.7969±0.1014 | **0.9708±0.0246** | 0.9975±0.0031 | 0.0277±0.0078 |
| ROS | 0.9646±0.0079 | 0.9892±0.0049 | 0.9734±0.0102 | 0.9812±0.0043 | 0.7867±0.1005 | 0.9701±0.0205 | 0.9976±0.0025 | 0.0314±0.0086 |
| SMOTE | 0.9633±0.0074 | 0.9906±0.0044 | 0.9707±0.0085 | 0.9805±0.004 | 0.8147±0.0894 | 0.9702±0.0200 | 0.9976±0.0026 | 0.0316±0.0071 |
| ADASYN | 0.9585±0.0112 | 0.9921±0.0046 | 0.9641±0.0139 | 0.9779±0.0062 | 0.8457±0.0926 | 0.9682±0.0202 | 0.9975±0.0026 | 0.0375±0.0112 |
| SMOTE_TL | 0.9630±0.0073 | 0.9910±0.0044 | 0.9700±0.0086 | 0.9804±0.0039 | 0.8226±0.0879 | 0.9693±0.0206 | 0.9975±0.0027 | 0.0320±0.0069 |
| SMOTE_ENN | 0.9479±0.0076 | 0.9962±0.0031 | 0.9489±0.0087 | 0.972±0.0042 | 0.9272±0.0591 | 0.9678±0.0213 | 0.9972±0.0030 | 0.0480±0.0075 |
| OUPS | 0.9598±0.0076 | 0.9926±0.0042 | 0.9650±0.0088 | 0.9786±0.0041 | 0.8560±0.0836 | 0.9690±0.0233 | 0.9975±0.0028 | 0.0345±0.0064 |
| PSM (1:4) | 0.9321±0.0058 | 0.9963±0.0024 | 0.9321±0.0050 | 0.9632±0.0032 | 0.9315±0.0454 | 0.9631±0.0134 | **0.9980±0.0010** | 0.0577±0.0051 |
| PSM (1:1) | 0.9257±0.0062 | **0.9983±0.0022** | 0.9235±0.0048 | 0.9595±0.0034 | **0.9680±0.0416** | 0.9337±0.0289 | 0.9976±0.0009 | **0.0711±0.0055** |
| Excluding demographic variables | | | | | | | | |
| Baseline | 0.9707±0.0063 | 0.9807±0.005 | 0.9888±0.0054 | 0.9847±0.0033 | 0.6099±0.1038 | 0.9464±0.0558 | 0.9941±0.0084 | 0.0242±0.0044 |
| RUS | 0.9464±0.0182 | 0.9942±0.0053 | 0.9493±0.0229 | 0.9711±0.0101 | 0.887±0.1074 | 0.9723±0.0177 | 0.9980±0.0018 | 0.0520±0.0119 |
| TL | 0.9716±0.0061 | 0.9849±0.0048 | 0.9853±0.0068 | 0.9851±0.0032 | 0.6978±0.0985 | 0.9517±0.0500 | 0.9945±0.0077 | 0.0239±0.0053 |
| OSS | **0.9718±0.0061** | 0.9848±0.0048 | **0.9856±0.0068** | **0.9852±0.0032** | 0.6949±0.0993 | 0.953±0.04620 | 0.9944±0.0080 | 0.0238±0.0051 |
| ENN | 0.9657±0.0073 | 0.9904±0.0041 | 0.9734±0.0085 | 0.9818±0.0039 | 0.8097±0.0839 | 0.9601±0.0358 | 0.9965±0.0045 | 0.0291±0.0077 |
| NCR | 0.9664±0.007 | 0.9898±0.0048 | 0.9748±0.0083 | 0.9822±0.0038 | 0.7977±0.0980 | 0.9583±0.0395 | 0.9961±0.0052 | 0.0278±0.0075 |
| ROS | 0.9541±0.0106 | 0.9924±0.0046 | 0.9591±0.0125 | 0.9754±0.0058 | 0.8521±0.0924 | 0.9649±0.0285 | 0.9973±0.0029 | 0.0386±0.0074 |
| SMOTE | 0.9507±0.0138 | 0.993±0.0052 | 0.9551±0.0178 | 0.9736±0.0076 | 0.8631±0.1032 | 0.9719±0.0186 | 0.9980±0.0019 | 0.0399±0.0075 |
| ADASYN | 0.9421±0.0165 | 0.9942±0.0056 | 0.9448±0.021 | 0.9687±0.0092 | 0.8878±0.1101 | 0.9697±0.0183 | 0.9979±0.0017 | 0.0491±0.0100 |
| SMOTE_TL | 0.9505±0.0136 | 0.9933±0.0048 | 0.9546±0.0175 | 0.9734±0.0076 | 0.8691±0.0968 | 0.9698±0.0231 | 0.9978±0.0024 | 0.0403±0.0076 |
| SMOTE_ENN | 0.9372±0.0103 | **0.9970±0.0029** | 0.9369±0.012 | 0.966±0.0058 | **0.9433±0.0552** | 0.9688±0.0193 | 0.9976±0.0019 | **0.0545±0.0075** |
| OUPS | 0.9479±0.0159 | 0.9935±0.0053 | 0.9516±0.0203 | 0.972±0.0089 | 0.8733±0.1050 | **0.9727±0.0170** | **0.9981±0.0017** | 0.0422±0.0083 |

**Supplementary Table S11. Classification Performance Metrics for the CVD Dataset With and Without Demographic Variables**

|  |  |  |  |  |  |  |  | (Mean ± SD) |
| --- | --- | --- | --- | --- | --- | --- | --- | --- |
| **Resampling Techniques** | **Accuracy** | **Precision** | **Recall** | **F1 Score** | **Specificity** | **AUROC** | **AUPRC** | **Brier Score** |
| Including demographic variables | | | | | | | | |
| Baseline | **0.9076±0.0005** | **0.5410±0.0114** | 0.1274±0.0034 | 0.2062±0.0051 | **0.9888±0.0004** | 0.8472±0.0028 | 0.3707±0.0057 | 0.0704±0.0003 |
| RUS | 0.7522±0.0031 | 0.2467±0.0032 | 0.7942±0.0059 | 0.3764±0.0042 | 0.7478±0.0030 | 0.8473±0.0028 | 0.3702±0.0057 | 0.1651±0.0012 |
| TL | 0.9070±0.0003 | 0.5219±0.0058 | 0.1558±0.0035 | 0.2400±0.0042 | 0.9852±0.0005 | 0.8472±0.0027 | 0.3706±0.0057 | 0.0707±0.0003 |
| OSS | 0.9072±0.0005 | 0.5248±0.0083 | 0.1528±0.0035 | 0.2366±0.0045 | 0.9856±0.0005 | 0.8472±0.0027 | 0.3707±0.0057 | 0.0706±0.0003 |
| ENN | 0.8904±0.0004 | 0.4085±0.0021 | 0.3646±0.006 | **0.3853±0.0037** | 0.9451±0.0009 | 0.8470±0.0027 | 0.3694±0.0056 | 0.0795±0.0004 |
| NCR | 0.8979±0.0004 | 0.4376±0.0020 | 0.2958±0.0044 | 0.3530±0.0029 | 0.9605±0.0008 | 0.8464±0.0027 | 0.3684±0.0057 | 0.0752±0.0005 |
| ROS | 0.7523±0.0028 | 0.2469±0.0030 | 0.7949±0.0054 | 0.3768±0.0040 | 0.7479±0.0027 | **0.8474±0.0027** | 0.3704±0.0057 | 0.165±0.0011 |
| SMOTE | 0.7566±0.0029 | 0.2484±0.0032 | 0.7819±0.0057 | 0.3770±0.0043 | 0.7540±0.0028 | 0.8461±0.0030 | 0.3696±0.0060 | 0.1622±0.0011 |
| ADASYN | 0.7443±0.0028 | 0.2412±0.0028 | 0.7989±0.005 | 0.3705±0.0038 | 0.7387±0.0027 | 0.8458±0.0030 | 0.3688±0.0061 | 0.1691±0.0012 |
| SMOTE_TL | 0.7566±0.0029 | 0.2484±0.0032 | 0.7819±0.0057 | 0.3770±0.0043 | 0.7540±0.0028 | 0.8461±0.0030 | 0.3696±0.0060 | 0.1622±0.0011 |
| SMOTE_ENN | 0.6715±0.0029 | 0.2071±0.0016 | **0.8798±0.0026** | 0.3353±0.0022 | 0.6498±0.0032 | 0.8470±0.0027 | 0.3697±0.0058 | **0.2387±0.0018** |
| OUPS | 0.7467±0.0029 | 0.2428±0.0029 | 0.7974±0.0058 | 0.3723±0.0039 | 0.7414±0.003 | 0.8458±0.0028 | 0.3675±0.0053 | 0.1678±0.0012 |
| PSM (1:4) | 0.9053±0.0005 | 0.4907±0.0101 | 0.1384±0.0054 | 0.2159±0.0073 | 0.9851±0.0005 | 0.8106±0.0029 | 0.3303±0.0065 | 0.0774±0.0004 |
| PSM (1:1) | 0.7444±0.0020 | 0.2195±0.0018 | 0.6708±0.0037 | 0.3308±0.0023 | 0.7520±0.0022 | 0.7824±0.0034 | 0.2977±0.0069 | 0.1795±0.0010 |
| Excluding demographic variables | | | | | | | | |
| Baseline | 0.9073±0.0005 | 0.5447±0.0146 | 0.0940±0.0045 | 0.1604±0.0072 | 0.9918±0.0002 | 0.8135±0.0029 | 0.3328±0.0059 | 0.0733±0.0004 |
| RUS | 0.7394±0.0021 | 0.2282±0.0019 | 0.7418±0.0047 | 0.3490±0.0026 | 0.7391±0.0022 | 0.8138±0.0028 | 0.3326±0.0058 | 0.1769±0.0010 |
| TL | **0.9067±0.0005** | **0.5203±0.0107** | 0.1177±0.0046 | 0.1920±0.0068 | 0.9887±0.0002 | 0.8136±0.0029 | **0.3327±0.0059** | 0.0735±0.0004 |
| OSS | 0.9067±0.0004 | 0.5215±0.0100 | 0.1149±0.0049 | 0.1882±0.0071 | **0.9890±0.0003** | 0.8136±0.0029 | **0.3327±0.0059** | 0.0735±0.0004 |
| ENN | 0.8931±0.0009 | 0.4026±0.0067 | 0.2793±0.0056 | 0.3298±0.0060 | 0.9569±0.0006 | 0.8137±0.0027 | 0.3317±0.0057 | 0.0802±0.0005 |
| NCR | 0.8984±0.0007 | 0.4270±0.0067 | 0.2292±0.0026 | 0.2983±0.0034 | 0.9680±0.0007 | 0.8130±0.0029 | 0.3310±0.0060 | 0.0771±0.0006 |
| ROS | 0.7396±0.0023 | 0.2284±0.0021 | 0.7416±0.0043 | **0.3492±0.0028** | 0.7394±0.0023 | **0.8139±0.0028** | 0.3327±0.0058 | 0.1768±0.0010 |
| SMOTE | 0.7407±0.0017 | 0.2280±0.0017 | 0.7347±0.0047 | 0.3480±0.0024 | 0.7413±0.0018 | 0.8117±0.0033 | 0.3311±0.0061 | 0.1751±0.0011 |
| ADASYN | 0.7280±0.0020 | 0.2216±0.0021 | 0.7512±0.0050 | 0.3422±0.0030 | 0.7256±0.0018 | 0.8113±0.0034 | 0.3304±0.0061 | 0.1814±0.0012 |
| SMOTE_TL | 0.7407±0.0017 | 0.2280±0.0017 | 0.7347±0.0047 | 0.3480±0.0024 | 0.7413±0.0018 | 0.8117±0.0033 | 0.3311±0.0061 | 0.1751±0.0011 |
| SMOTE_ENN | 0.6577±0.0019 | 0.1935±0.0014 | **0.8313±0.0043** | 0.3139±0.0021 | 0.6397±0.0018 | **0.8139±0.0028** | 0.3323±0.0055 | **0.2472±0.0013** |
| OUPS | 0.7380±0.0021 | 0.2269±0.0021 | 0.7404±0.0049 | 0.3474±0.0029 | 0.7377±0.0020 | 0.8120±0.0028 | 0.3304±0.0055 | 0.1783±0.0010 |

Supplementary Table S12 reports the detailed classification performance metrics (mean ± SD) for the HypoT dataset based on variable distribution, corresponding to the results presented in Fig.~7 and Section~3.4 (Comparison of Classification Performance Based on Variable Distribution). Performance metrics were computed across 10 random seeds with 5-fold cross-validation under different variable distribution settings. In Supplementary Tables S12, the best-performing result for each metric across resampling strategies is highlighted in bold.

**Supplementary Table S12. Classification Performance Metrics for the HypoT Dataset based on variable distribution.**

|  |  |  |  |  |  |  |  | (Mean ± SD) |
| --- | --- | --- | --- | --- | --- | --- | --- | --- |
| **Resampling Techniques** | **Accuracy** | **Precision** | **Recall** | **F1 Score** | **Specificity** | **AUROC** | **AUPRC** | **Brier Score** |
| Matching using Sex | | | | | | | | |
| Baseline | 0.9696±0.0054 | 0.9806±0.0052 | **0.9876±0.0058** | 0.9841±0.0028 | 0.6089±0.1083 | 0.9500±0.0464 | 0.9947±0.0070 | 0.0247±0.0041 |
| RUS | 0.9464±0.0198 | 0.9936±0.0053 | 0.9500±0.0246 | 0.9711±0.0110 | 0.8743±0.1080 | **0.9698±0.0194** | **0.9978±0.0021** | 0.0526±0.0123 |
| TL | 0.9707±0.0052 | 0.9848±0.0047 | 0.9844±0.0068 | 0.9846±0.0027 | 0.6959±0.0983 | 0.9549±0.0403 | 0.9956±0.0057 | 0.0246±0.0050 |
| OSS | **0.9708±0.0052** | 0.9844±0.0047 | 0.9849±0.0068 | **0.9846±0.0028** | 0.6875±0.0978 | 0.9562±0.0385 | 0.9959±0.0053 | 0.0246±0.0049 |
| ENN | 0.9646±0.0072 | 0.9902±0.0038 | 0.9725±0.0089 | 0.9812±0.0039 | 0.8061±0.0785 | 0.9660±0.0245 | 0.9975±0.0023 | 0.0299±0.0075 |
| NCR | 0.9658±0.0071 | 0.9892±0.0042 | 0.9748±0.0086 | 0.9819±0.0038 | 0.7859±0.0862 | 0.9634±0.0285 | 0.9972±0.0028 | 0.0285±0.0072 |
| ROS | 0.9573±0.0074 | 0.9901±0.0051 | 0.9649±0.0094 | 0.9773±0.004 | 0.8056±0.1025 | 0.9631±0.0287 | 0.9972±0.0029 | 0.0374±0.0058 |
| SMOTE | 0.9560±0.0094 | 0.9918±0.0044 | 0.9618±0.0122 | 0.9765±0.0052 | 0.8405±0.0881 | 0.9679±0.0193 | 0.9978±0.0019 | 0.0367±0.0059 |
| ADASYN | 0.9477±0.0129 | 0.9934±0.0047 | 0.9515±0.0166 | 0.9719±0.0072 | 0.8717±0.0941 | 0.9653±0.0195 | 0.9976±0.0019 | 0.0449±0.0086 |
| SMOTE_TL | 0.9555±0.0090 | 0.9921±0.0043 | 0.9610±0.0116 | 0.9763±0.0049 | 0.8452±0.0856 | 0.9664±0.0213 | 0.9976±0.0021 | 0.0372±0.0059 |
| SMOTE_ENN | 0.9411±0.0082 | **0.9963±0.0027** | 0.9417±0.0095 | 0.9682±0.0046 | 0.9291±0.0519 | 0.9681±0.0186 | 0.9975±0.002 | 0.0522±0.0066 |
| OUPS | 0.9539±0.0106 | 0.9920±0.0040 | 0.9594±0.0134 | 0.9753±0.0059 | 0.8445±0.0818 | 0.9684±0.0186 | 0.9977±0.0019 | 0.0386±0.0063 |
| PSM (1:4) | 0.9603±0.0119 | 0.5921±0.1267 | 0.7647±0.1175 | 0.6520±0.0715 | **0.9701±0.0151** | 0.9694±0.0218 | 0.6680±0.1045 | 0.0312±0.0082 |
| PSM (1:1) | 0.3573±0.1191 | 0.0755±0.063 | 0.9365±0.044 | 0.1345±0.0802 | 0.3283±0.1254 | 0.9002±0.0601 | 0.5842±0.1063 | **0.4996±0.1413** |
| Matching using Age | | | | | | | | |
| Baseline | 0.9708±0.0047 | 0.9809±0.0052 | **0.9887±0.0056** | 0.9848±0.0025 | 0.6141±0.1090 | 0.9537±0.0503 | 0.9950±0.0076 | 0.0237±0.0039 |
| RUS | 0.9489±0.0169 | 0.9939±0.0054 | 0.9523±0.0216 | 0.9725±0.0094 | 0.8811±0.1090 | 0.9714±0.0208 | **0.9979±0.0024** | 0.0508±0.0116 |
| TL | **0.9720±0.0052** | 0.9854±0.0051 | 0.9853±0.0072 | **0.9853±0.0028** | 0.7068±0.1061 | 0.9589±0.0439 | 0.9958±0.0064 | 0.0233±0.0047 |
| OSS | 0.9720±0.0050 | 0.9852±0.0051 | 0.9855±0.0074 | 0.9853±0.0026 | 0.7024±0.1056 | 0.9615±0.0381 | 0.9966±0.0045 | 0.0233±0.0045 |
| ENN | 0.9642±0.0070 | 0.9906±0.0044 | 0.9717±0.0089 | 0.9810±0.0038 | 0.8144±0.0885 | 0.9721±0.0215 | **0.9979±0.0023** | 0.0302±0.0073 |
| NCR | 0.9662±0.0079 | 0.9896±0.0047 | 0.9748±0.0091 | 0.9821±0.0042 | 0.7950±0.0954 | 0.9698±0.0255 | 0.9977±0.0026 | 0.0282±0.0076 |
| ROS | 0.9633±0.0086 | 0.9895±0.0051 | 0.9718±0.0112 | 0.9805±0.0047 | 0.7922±0.1048 | 0.9711±0.0202 | 0.9977±0.0024 | 0.0320±0.0088 |
| SMOTE | 0.9605±0.0078 | 0.9917±0.0046 | 0.9667±0.0095 | 0.9790±0.0042 | 0.8375±0.0914 | 0.9720±0.0184 | 0.9978±0.0023 | 0.0338±0.0071 |
| ADASYN | 0.9537±0.0124 | 0.9934±0.0044 | 0.9578±0.0153 | 0.9752±0.0069 | 0.8713±0.0882 | 0.9707±0.0182 | 0.9978±0.0022 | 0.0408±0.0106 |
| SMOTE_TL | 0.9596±0.0079 | 0.9919±0.0045 | 0.9656±0.0096 | 0.9785±0.0043 | 0.8410±0.0902 | 0.9700±0.0205 | 0.9976±0.0027 | 0.0345±0.0069 |
| SMOTE_ENN | 0.9455±0.0073 | **0.9965±0.0029** | 0.9461±0.0084 | 0.9706±0.0041 | 0.9323±0.0557 | 0.9674±0.0212 | 0.9973±0.0027 | 0.0497±0.0071 |
| OUPS | 0.9566±0.0092 | 0.9937±0.0044 | 0.9606±0.0108 | 0.9768±0.0050 | 0.8765±0.0883 | 0.9710±0.0228 | 0.9978±0.0024 | 0.0369±0.0067 |
| PSM (1:4) | 0.9657±0.0065 | 0.651±0.1143 | 0.7438±0.1589 | 0.6708±0.0491 | **0.9768±0.0128** | **0.9735±0.0177** | 0.7074±0.0861 | 0.0280±0.0049 |
| PSM (1:1) | 0.1495±0.0548 | 0.0523±0.0027 | 0.9801±0.0331 | 0.0994±0.0049 | 0.1078±0.0586 | 0.8123±0.1265 | 0.4217±0.1991 | **0.7550±0.1006** |
| Matching using Sex while excluding Age | | | | | | | | |
| PSM (1:4) | **0.9603±0.0119** | **0.5921±0.1267** | 0.7647±0.1175 | **0.6520±0.0715** | **0.9701±0.0151** | **0.9694±0.0218** | **0.6680±0.1045** | 0.0312±0.0082 |
| PSM (1:1) | 0.3573±0.1191 | 0.0755±0.0630 | **0.9365±0.0440** | 0.1345±0.0802 | 0.3283±0.1254 | 0.9002±0.0601 | 0.5842±0.1063 | **0.4996±0.1413** |
| Matching using Age while excluding Sex | | | | | | | | |
| PSM (1:4) | **0.9657±0.0065** | **0.6510±0.1143** | 0.7438±0.1589 | **0.6708±0.0491** | **0.9768±0.0128** | **0.9735±0.0177** | **0.7074±0.0861** | 0.0280±0.0049 |
| PSM (1:1) | 0.1495±0.0548 | 0.0523±0.0027 | **0.9801±0.0331** | 0.0994±0.0049 | 0.1078±0.0586 | 0.8123±0.1265 | 0.4217±0.1991 | **0.7550±0.1006** |


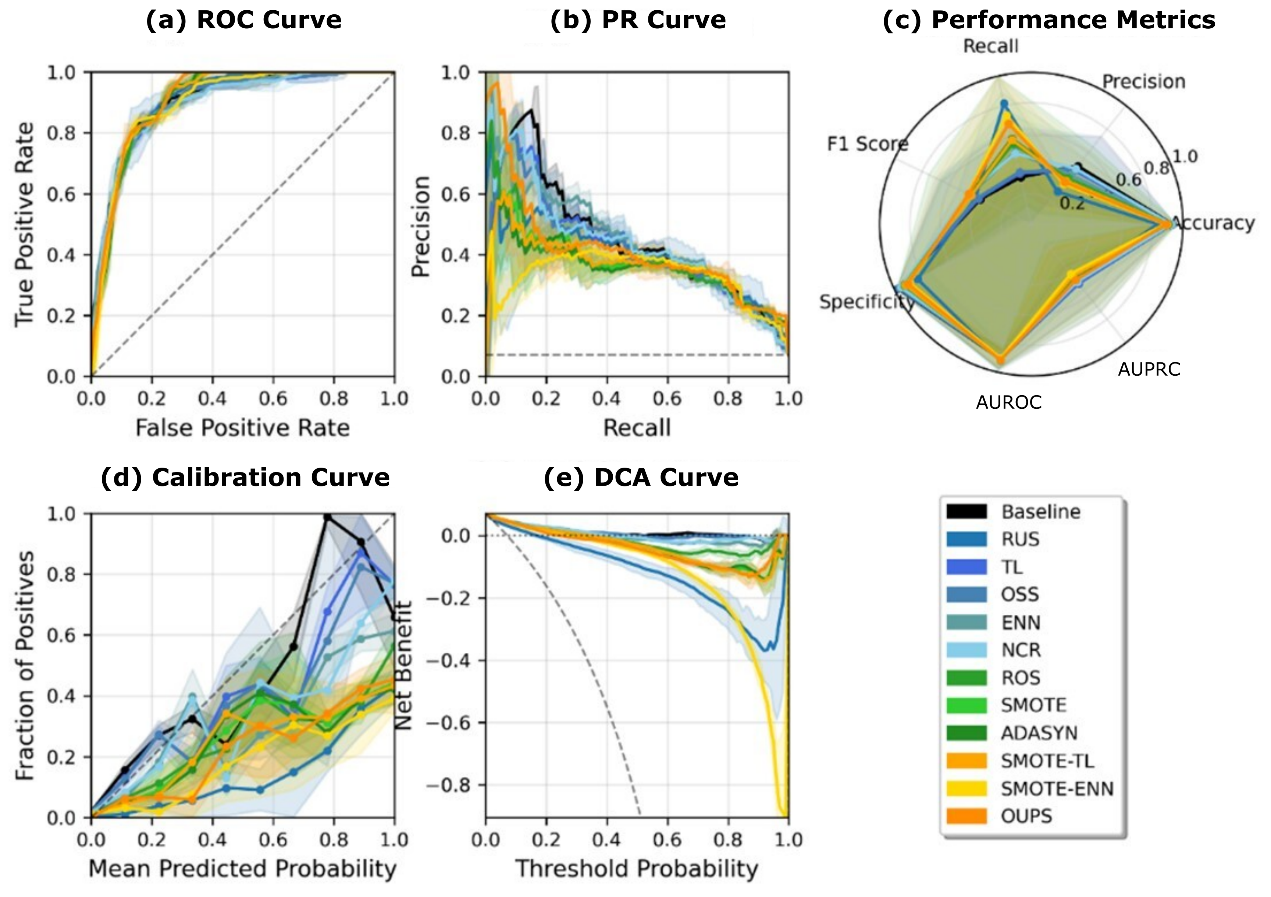


Supplementary Figure S1. Comprehensive performance comparison of resampling strategies on the RPD dataset without demographic variables, evaluated in terms of discrimination, calibration, and clinical utility, corresponding to the analyses presented in Fig. 4. Panels show (a) ROC Curves, (b) PR Curves, (c) aggregated classification metrics, (d) Calibration Curves, and (e) Decision Curve Analysis (DCA).


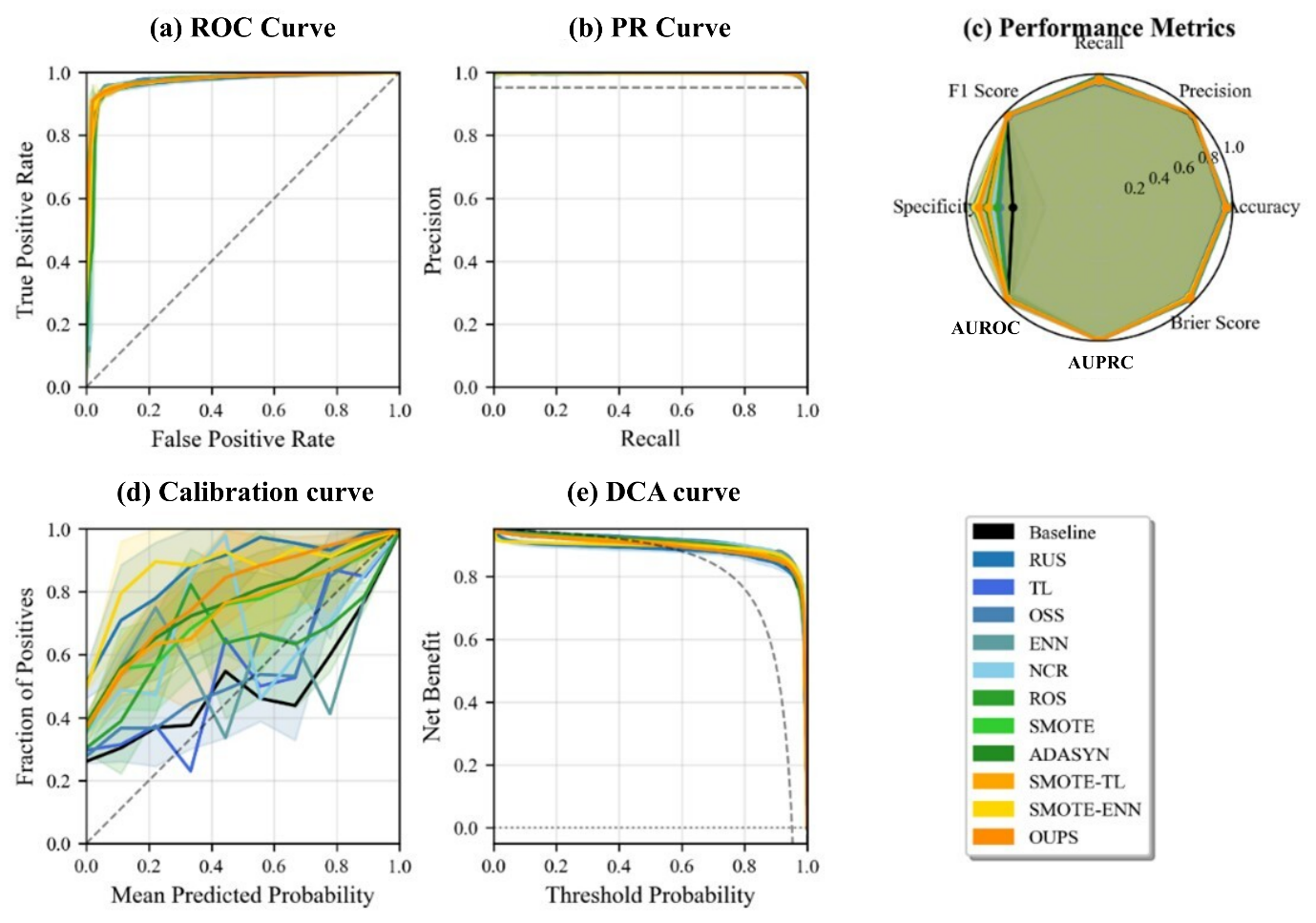


Supplementary Figure S2. Comprehensive performance comparison of resampling strategies on the HypoT dataset without demographic variables, evaluated in terms of discrimination, calibration, and clinical utility, corresponding to the analyses presented in Fig. 5. Panels show (a) ROC Curves, (b) PR Curves, (c) aggregated classification metrics, (d) Calibration Curves, and (e) Decision Curve Analysis (DCA).


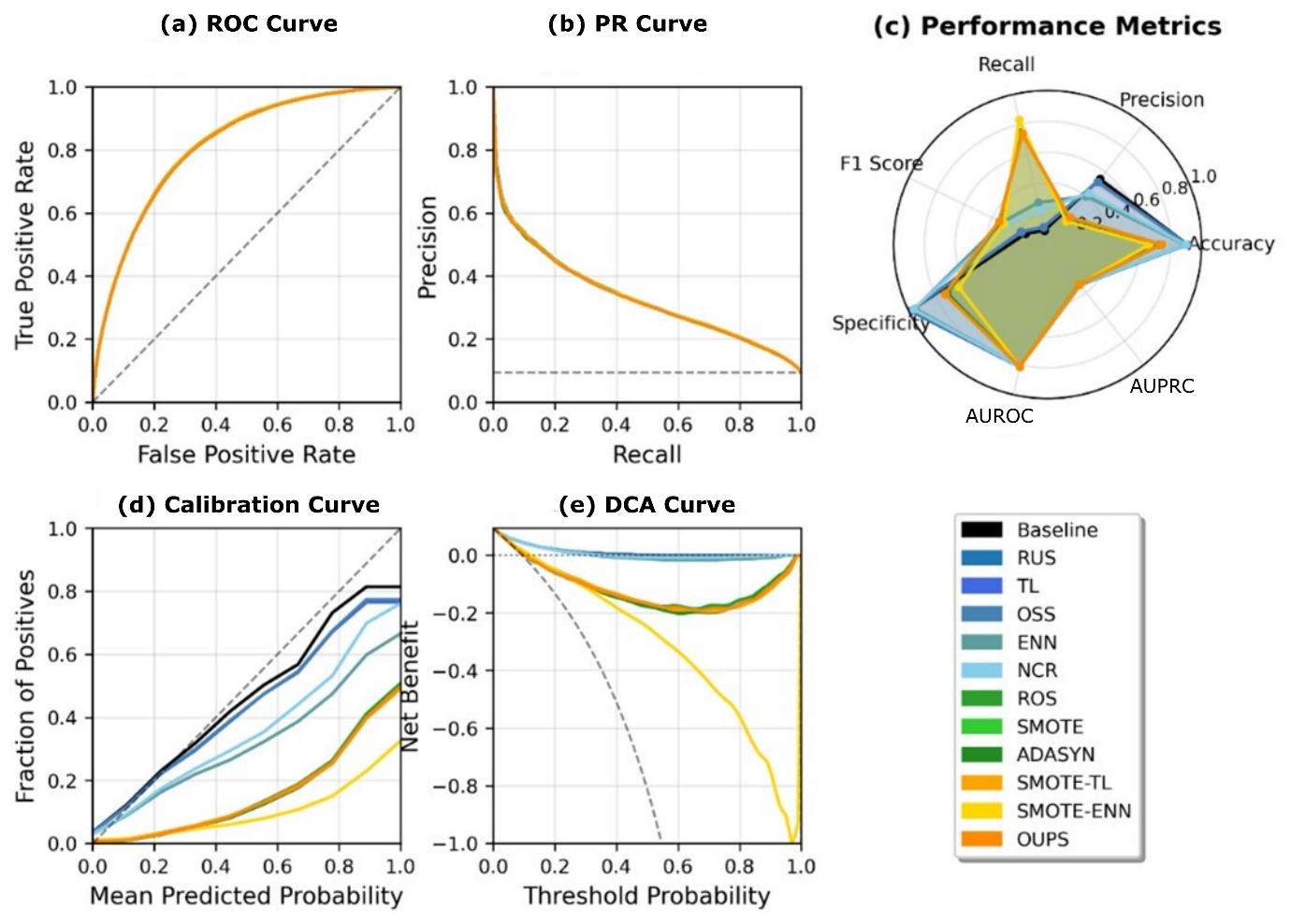


Supplementary Figure S3. Comprehensive performance comparison of resampling strategies on the CVD dataset without demographic variables, evaluated in terms of discrimination, calibration, and clinical utility, corresponding to the analyses presented in Fig. 6. Panels show (a) ROC Curves, (b) PR Curves, (c) aggregated classification metrics, (d) Calibration Curves, and (e) Decision Curve Analysis (DCA).


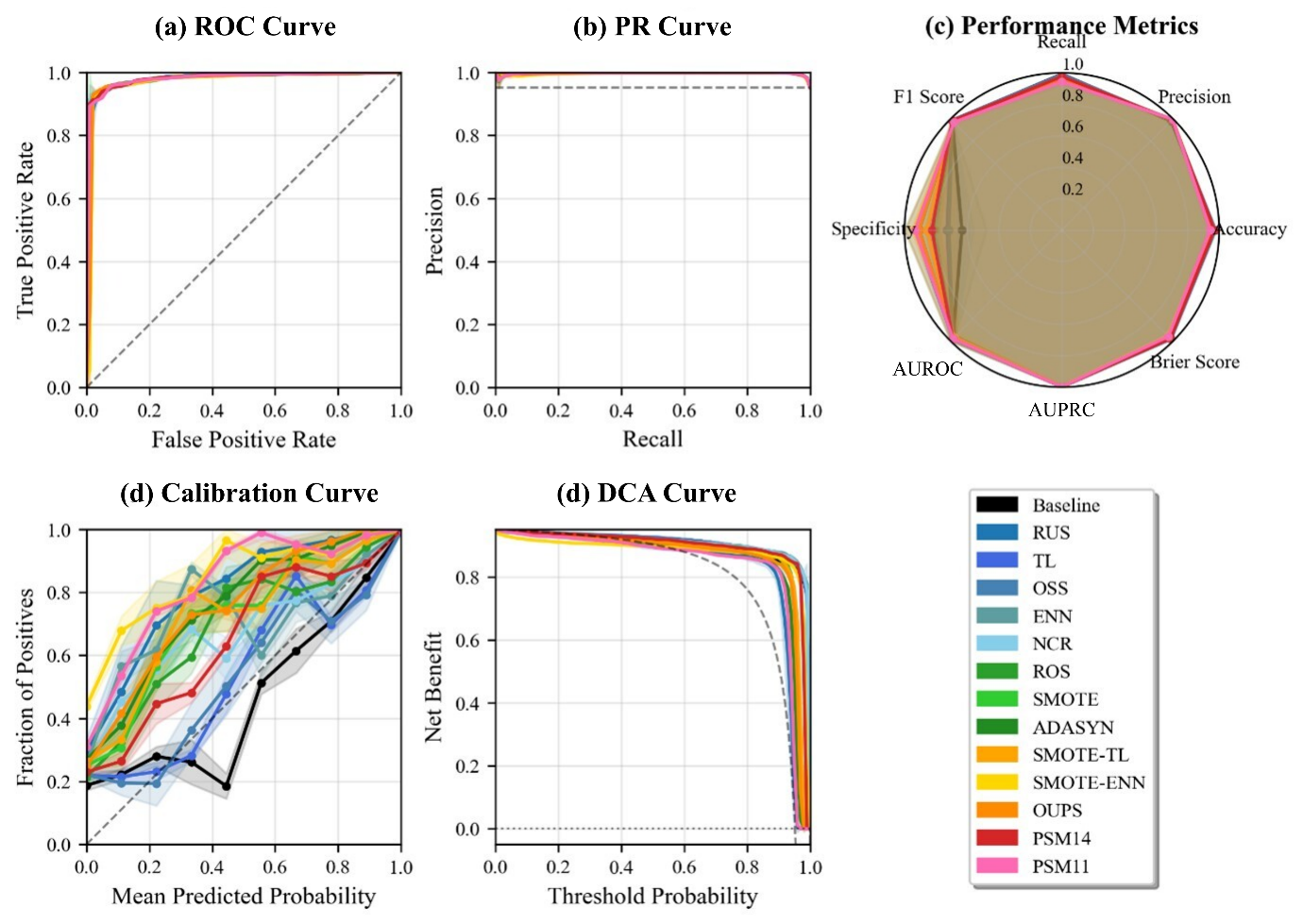


Supplementary Figure S4. Comprehensive performance comparison of resampling strategies on the HypoT dataset under the matching using Sex condition for PSM, evaluated in terms of discrimination, calibration, and clinical utility, corresponding to the analyses presented for matching using Sex in Fig.7. The PSM results are identical to those shown in Fig.7, whereas the results of other resampling techniques are included for comparative purposes under the same experimental setting, corresponding to the matching using Age while excluding Sex condition in Fig.7. Panels show (a) ROC Curves, (b) PR Curves, (c) aggregated classification metrics, (d) Calibration Curves, and (e) Decision Curve Analysis (DCA).


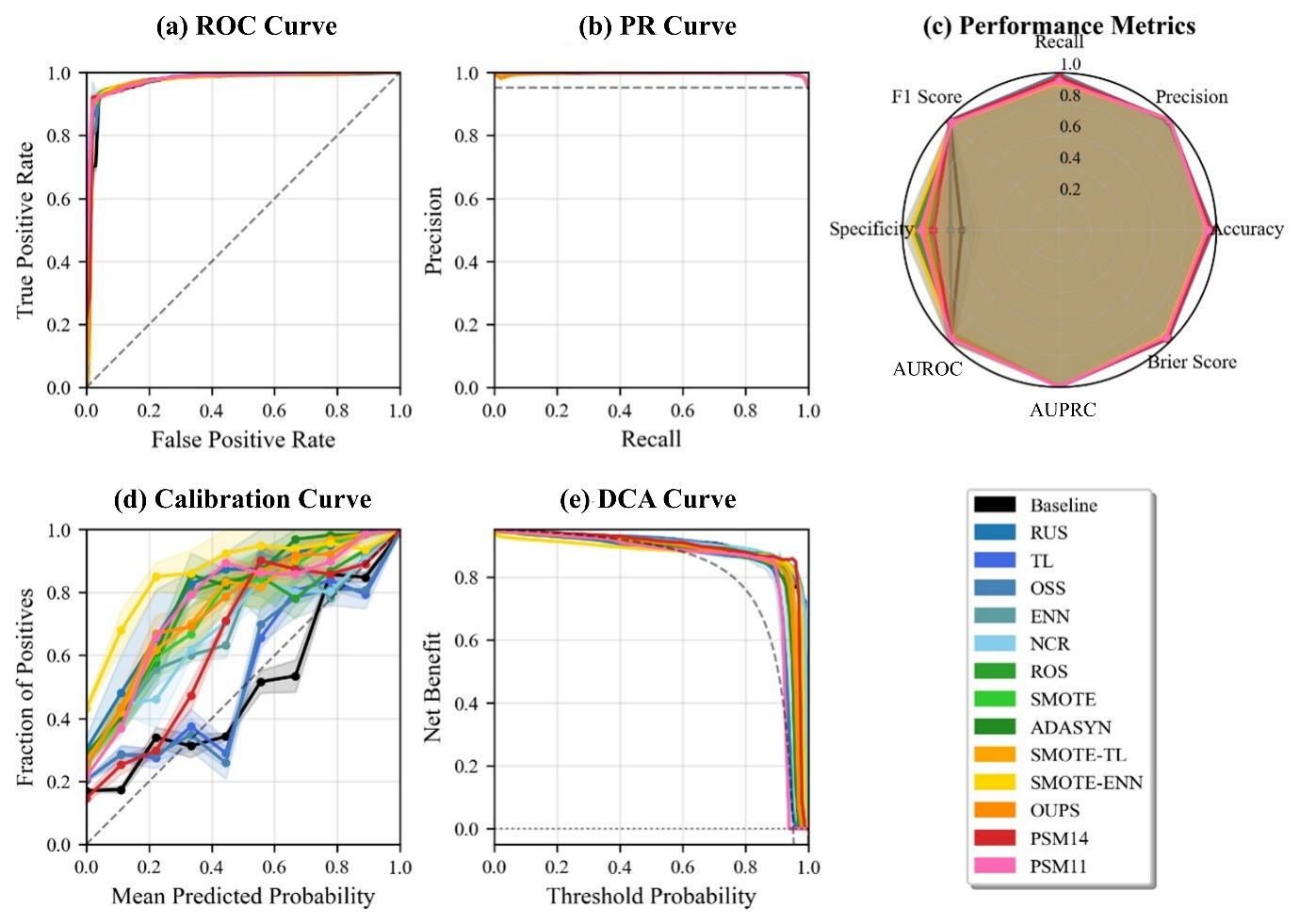


Supplementary Figure S5. Comprehensive performance comparison of resampling strategies on the HypoT dataset under the matching using Age condition for PSM, evaluated in terms of discrimination, calibration, and clinical utility, corresponding to the analyses presented for matching using Age in Fig.7. The PSM results are identical to those shown in Fig.7, whereas the results of other resampling techniques are included for comparative purposes under the same experimental setting, corresponding to the matching using Sex while excluding Age condition in Fig.7. Panels show (a) ROC Curves, (b) PR Curves, (c) aggregated classification metrics, (d) Calibration Curves, and (e) Decision Curve Analysis (DCA).


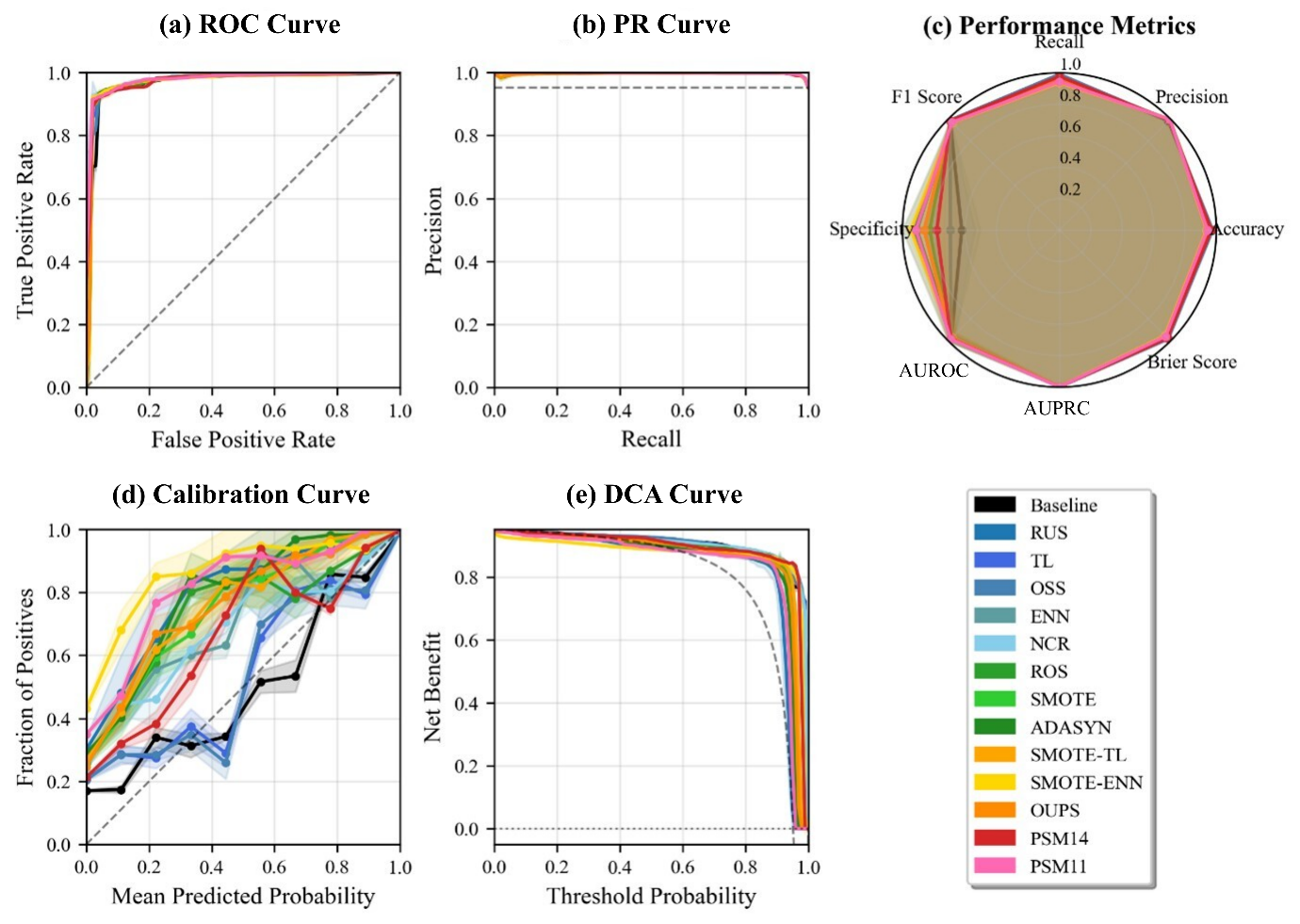


Supplementary Figure S6. Comprehensive performance comparison of resampling strategies on the HypoT dataset under the matching using Sex excluding Age, evaluated in terms of discrimination, calibration, and clinical utility, corresponding to the analyses presented for matching using Sex excluding Age in Fig.7. Panels show (a) ROC Curves, (b) PR Curves, (c) aggregated classification metrics, (d) Calibration Curves, and (e) Decision Curve Analysis (DCA).


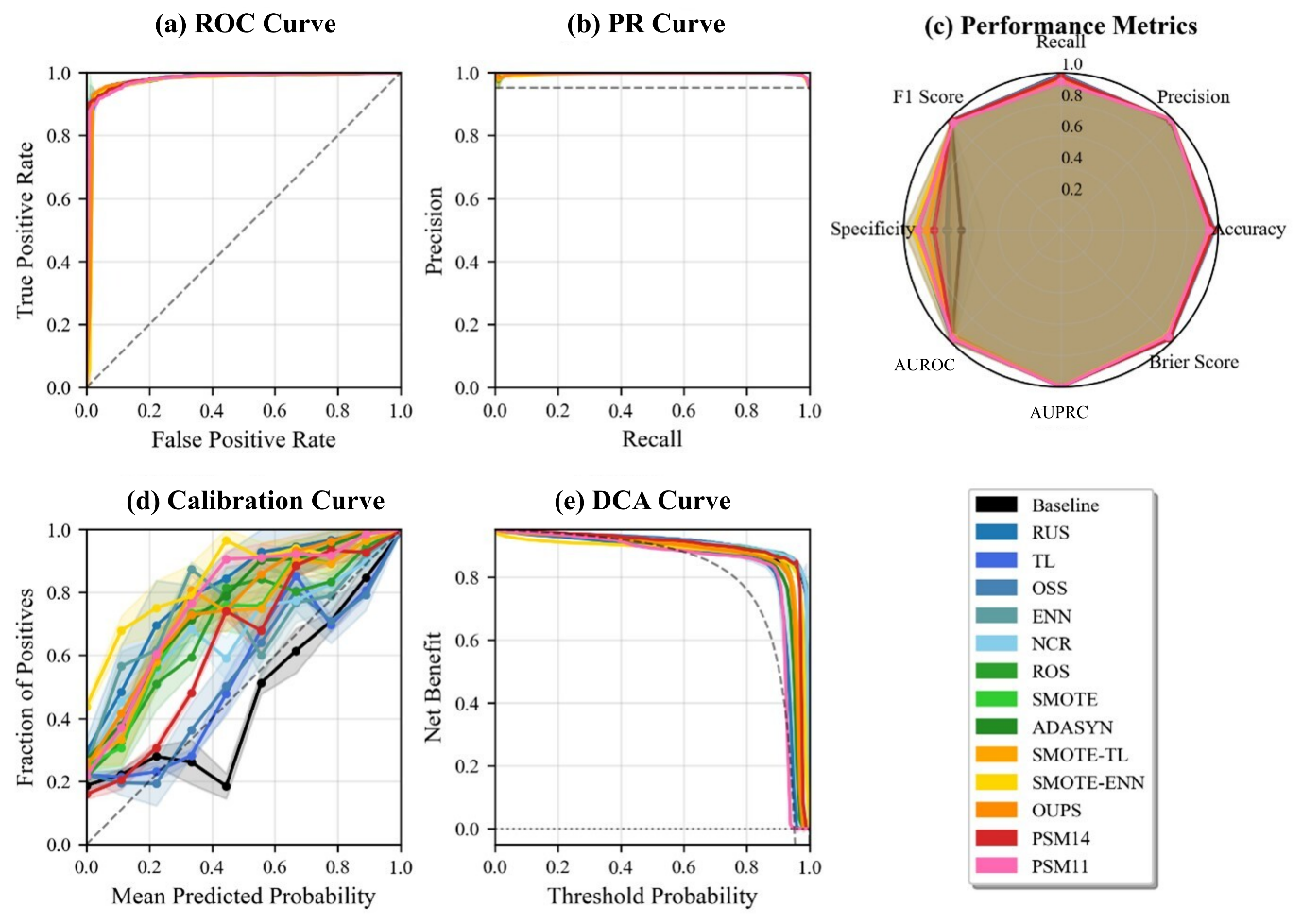


Supplementary Figure S7. Comprehensive performance comparison of resampling strategies on the HypoT dataset under the matching using Age excluding Sex, evaluated in terms of discrimination, calibration, and clinical utility, corresponding to the analyses presented for matching using Age excluding Sex in Fig.7. Panels show (a) ROC Curves, (b) PR curves, (c) aggregated classification metrics, (d) Calibration Curves, and (e) Decision Curve Analysis (DCA).

# Supplementary Material S7. TRIPOD+AI Checklist

| Section/Topic | Item | Development / evaluation^[[1]](#footnote-1)^ | Checklist item | Reported on page |
| --- | --- | --- | --- | --- |
| **TITLE** | | | |  |
| Title | 1 | D;E | Identify the study as developing or evaluating the performance of a multivariable prediction model, the target population, and the outcome to be predicted | Page 1 |
| **ABSTRACT** | | | | |
| Abstract | 2 | D;E | See TRIPOD+AI for Abstracts checklist | Page 1 |
| **INTRODUCTION** | | | | |
| Background | 3a | D;E | Explain the healthcare context (including whether diagnostic or prognostic) and rationale for developing or evaluating the prediction model, including references to existing models | Page 2-3 |
|  | 3b | D;E | Describe the target population and the intended purpose of the prediction model in the context of the care pathway, including its intended users (e.g., healthcare professionals, patients, public) | Page 3-4 |
|  | 3c | D;E | Describe any known health inequalities between sociodemographic groups | Table 2, Supplementary Material S1 |
| Objectives | 4 | D;E | Specify the study objectives, including whether the study describes the development or validation of a prediction model (or both) | Page 3 |
| **METHODS** | | | | |
| Data | 5a | D;E | Describe the sources of data separately for the development and evaluation datasets (e.g., randomized trial, cohort, routine care or registry data), the rationale for using these data, and representativeness of the data | Page 3-4, 19 |
|  | 5b | D;E | Specify the dates of the collected participant data, including start and end of participant accrual; and, if applicable, end of follow-up | Not Applicable |
| Participants | 6a | D;E | Specify key elements of the study setting (e.g., primary care, secondary care, general population) including the number and location of centers | Not Applicable |
|  | 6b | D;E | Describe the eligibility criteria for study participants | Not Applicable |
|  | 6c | D;E | Give details of any treatments received, and how they were handled during model development or evaluation, if relevant | Not Applicable |
| Data preparation | 7 | D;E | Describe any data pre-processing and quality checking, including whether this was similar across relevant sociodemographic groups | Page 3-4,  Table 2, |
| Outcome | 8a | D;E | Clearly define the outcome that is being predicted and the time horizon, including how and when assessed, the rationale for choosing this outcome, and whether the method of outcome assessment is consistent across sociodemographic groups | Not Applicable |
|  | 8b | D;E | If outcome assessment requires subjective interpretation, describe the qualifications and demographic characteristics of the outcome assessors | Not Applicable |
|  | 8c | D;E | Report any actions to blind assessment of the outcome to be predicted | Not Applicable |
| Predictors | 9a | D | Describe the choice of initial predictors (e.g., literature, previous models, all available predictors) and any pre-selection of predictors before model building | Not Applicable |
|  | 9b | D;E | Clearly define all predictors, including how and when they were measured (and any actions to blind assessment of predictors for the outcome and other predictors) | Page 3-4 |
|  | 9c | D;E | If predictor measurement requires subjective interpretation, describe the qualifications and demographic characteristics of the predictor assessors | Not Applicable |
| Sample size | 10 | D;E | Explain how the study size was arrived at (separately for development and evaluation), and justify that the study size was sufficient to answer the research question. Include details of any sample size calculation | Not Applicable |
| Missing data | 11 | D;E | Describe how missing data were handled. Provide reasons for omitting any data | Page 3-4 |
| Analytical methods | 12a | D | Describe how the data were used (e.g., for development and evaluation of model performance) in the analysis, including whether the data were partitioned, considering any sample size requirements | Page 6-7 |
|  | 12b | D | Depending on the type of model, describe how predictors were handled in the analyses (functional form, rescaling, transformation, or any standardization). | Not Applicable |
|  | 12c | D | Specify the type of model, rationale^[[2]](#footnote-2)^, all model-building steps, including any hyperparameter tuning, and method for internal validation | Page 6-7, Supplementary Material S2 |
|  | 12d | D;E | Describe if and how any heterogeneity in estimates of model parameter values and model performance was handled and quantified across clusters (e.g., hospitals, countries). See TRIPOD-Cluster for additional considerations^[[3]](#footnote-3)^ | Not Applicable |
|  | 12e | D;E | Specify all measures and plots used (and their rationale) to evaluate model performance (e.g., discrimination, calibration, clinical utility) and, if relevant, to compare multiple models | Supplementary Material S4 |
|  | 12f | E | Describe any model updating (e.g., recalibration) arising from the model evaluation, either overall or for particular sociodemographic groups or settings | Not Applicable |
|  | 12g | E | For model evaluation, describe how the model predictions were calculated (e.g., formula, code, object, application programming interface) | Supplementary Material S3 |
| Class imbalance | 13 | D;E | If class imbalance methods were used, state why and how this was done, and any subsequent methods to recalibrate the model or the model predictions | Page 4-5 |
| Fairness | 14 | D;E | Describe any approaches that were used to address model fairness and their rationale | Supplementary Material S3 |
| Model output | 15 | D | Specify the output of the prediction model (e.g., probabilities, classification). Provide details and rationale for any classification and how the thresholds were identified | Supplementary Material S3 |
| Training versus evaluation | 16 | D;E | Identify any differences between the development and evaluation data in healthcare setting, eligibility criteria, outcome, and predictors | Not Applicable |
| Ethical approval | 17 | D;E | Name the institutional research board or ethics committee that approved the study and describe the participant-informed consent or the ethics committee waiver of informed consent | Page 6 |
| **OPEN SCIENCE** | | | | |
| Funding | 18a | D;E | Give the source of funding and the role of the funders for the present study | Page 12 |
| Conflicts of interest | 18b | D;E | Declare any conflicts of interest and financial disclosures for all authors | Page 12 |
| Protocol | 18c | D;E | Indicate where the study protocol can be accessed or state that a protocol was not prepared | Not Applicable |
| Registration | 18d | D;E | Provide registration information for the study, including register name and registration number, or state that the study was not registered | Not Applicable |
| Data sharing | 18e | D;E | Provide details of the availability of the study data | Page 19 |
| Code sharing | 18f | D;E | Provide details of the availability of the analytical code^[[4]](#footnote-4)^ | Page 19 |
| **PATIENT & PUBLIC INVOLVEMENT** | | | | |
| Patient & Public Involvement | 19 | D;E | Provide details of any patient and public involvement during the design, conduct, reporting, interpretation, or dissemination of the study or state no involvement. | Not Applicable |
| **RESULTS** | | | | |
| Participants | 20a | D;E | Describe the flow of participants through the study, including the number of participants with and without the outcome and, if applicable, a summary of the follow-up time. A diagram may be helpful. | Not Applicable |
|  | 20b | D;E | Report the characteristics overall and, where applicable, for each data source or setting, including the key dates, key predictors (including demographics), treatments received, sample size, number of outcome events, follow-up time, and amount of missing data. A table may be helpful. Report any differences across key demographic groups. | Not Applicable |
|  | 20c | E | For model evaluation, show a comparison with the development data of the distribution of important predictors (demographics, predictors, and outcome). | Not Applicable |
| Model development | 21 | D;E | Specify the number of participants and outcome events in each analysis (e.g., for model development, hyperparameter tuning, model evaluation) | Figure 1-2, Supplementary Material S5 |
| Model specification | 22 | D | Provide details of the full prediction model (e.g., formula, code, object, application programming interface) to allow predictions in new individuals and to enable third-party evaluation and implementation, including any restrictions to access or re-use (e.g., freely available, proprietary)^[[5]](#footnote-5)^ | Supplementary Material S3 |
| Model performance | 23a | D;E | Report model performance estimates with confidence intervals, including for any key subgroups (e.g., sociodemographic). Consider plots to aid presentation. | Figure 3-7 |
|  | 23b | D;E | If examined, report results of any heterogeneity in model performance across clusters. See TRIPOD Cluster for additional details^3^ | Not Applicable |
| Model updating | 24 | E | Report the results from any model updating, including the updated model and subsequent performance | Not Applicable |
| **DISCUSSION** | | | | |
| Interpretation | 25 | D;E | Give an overall interpretation of the main results, including issues of fairness in the context of the objectives and previous studies | Page 13-17 |
| Limitations | 26 | D;E | Discuss any limitations of the study (such as a non-representative sample, sample size, overfitting, missing data) and their effects on any biases, statistical uncertainty, and generalizability | Page 17 |
| Usability of the model in the context of current care | 27a | D | Describe how poor quality or unavailable input data (e.g., predictor values) should be assessed and handled when implementing the prediction model | Page 13-17 |
|  | 27b | D | Specify whether users will be required to interact in the handling of the input data or use of the model, and what level of expertise is required of users | Not Applicable |
|  | 27c | D;E | Discuss any next steps for future research, with a specific view to applicability and generalizability of the model | Page 17 |

1. D=items relevant only to the development of a prediction model; E=items relating solely to the evaluation of a prediction model; D;E=items applicable to both the development and evaluation of a prediction model. [↑](#footnote-ref-1)
2. Separately for all model building approaches. [↑](#footnote-ref-2)
3. TRIPOD-Cluster is a checklist of reporting recommendations for studies developing or validating models that explicitly account for clustering or explore heterogeneity in model performance (eg, at different hospitals or centres). Debray et al, BMJ 2023; 380: e071018 [DOI: 10.1136/bmj-2022-071018]. [↑](#footnote-ref-3)
4. This relates to the analysis code, for example, any data cleaning, feature engineering, model building, evaluation. [↑](#footnote-ref-4)
5. This relates to the code to implement the model to get estimates of risk for a new individual. [↑](#footnote-ref-5)
